# Linking dietary fiber to human malady through cumulative profiling of microbiota disturbance

**DOI:** 10.1002/imt2.70004

**Published:** 2025-02-19

**Authors:** Xin Zhang, Huan Liu, Yu Li, Yanlong Wen, Tianxin Xu, Chen Chen, Shuxia Hao, Jielun Hu, Shaoping Nie, Fei Gao, Gengjie Jia

**Affiliations:** ^1^ Genome Analysis Laboratory of the Ministry of Agriculture and Rural Affairs, Agricultural Genomics Institute at Shenzhen Chinese Academy of Agricultural Sciences Shenzhen China; ^2^ State Key Laboratory of Food Science and Resources China‐Canada Joint Lab of Food Science and Technology (Nanchang), Key Laboratory of Bioactive Polysaccharides of Jiangxi Province, Nanchang University Nanchang China; ^3^ Department of Computer Science and Engineering The Chinese University of Hong Kong Hong Kong China; ^4^ Comparative Pediatrics and Nutrition, Department of Veterinary and Animal Sciences, Faculty of Health and Medical Sciences University of Copenhagen Copenhagen Denmark

**Keywords:** dietary fiber, disturbance score, human disease, integrative and comparative computation, microbiota disturbance, systematic analysis

## Abstract

Dietary fiber influences the composition and metabolic activity of microbial communities, impacting disease development. Current understanding of the intricate fiber‐microbe‐disease tripartite relationship remains fragmented and elusive, urging a systematic investigation. Here, we focused on microbiota disturbance as a robust index to mitigate various confounding factors and developed the Bio‐taxonomic Hierarchy Weighted Aggregation (BHWA) algorithm to integrate multi‐taxonomy microbiota disturbance data, thereby illuminating the complex relationships among dietary fiber, microbiota, and disease. By leveraging microbiota disturbance similarities, we (1) classified 32 types of dietary fibers into six functional subgroups, revealing correlations with fiber solubility; (2) established associations among 161 diseases, uncovering shared microbiota disturbance patterns that explain disease co‐occurrence (e.g., type II diabetes and kidney diseases) and distinct microbiota patterns that discern symptomatically similar diseases (e.g., inflammatory bowel disease and irritable bowel syndrome); (3) designed a body‐site‐specific microbiota disturbance scoring scheme, computing a disturbance score (*DS*) for each disease and highlighting the pronounced capacity of Crohn's disease to disturb gut microbiota (*DS* = 14.01) in contrast with food allergy's minimal capacity (*DS* = 0.74); (4) identified 1659 fiber‐disease associations, predicting the potential of dietary fiber to modulate specific microbiota changes associated with diseases of interest; (5) established murine models of inflammatory bowel disease to validate the preventive and therapeutic effects of arabinoxylan that notably perturbed the *Bacteroidetes* and *Firmicutes* phyla, as well as the *Bacteroidetes* and *Lactobacillus* genera, aligning with our model predictions. To enhance data accessibility and facilitate targeted dietary intervention development, we launched an interactive webtool—mDiFiBank at https://mdifibank.org.cn/.

## Highlights

- Our study explored the intricate relationships between dietary fibers, microbiota disturbances, and disease states, introducing Bio‐taxonomic Hierarchy Weighted Aggregation algorithm to analyze these interactions across taxonomic levels and body sites.
- By treating microbiota disturbances as key intermediaries, we established connections between dietary fiber intake and health outcomes, generating a candidate list of dietary fibers with therapeutic potential.
- We launched an interactive webtool—mDiFiBank (https://mdifibank.org.cn/)—to enhance data accessibility and facilitate the development of targeted dietary interventions.

## Graphical abstract

We have intensively searched existing literature repositories and databases. Using microbial disturbance as a robust index, we systematically revealed the complex relationships among dietary fiber, microbiota, and diseases. Key challenges include quantifying the multi‐taxonomic microbiota disturbances induced by dietary fibers or disease onset, as well as determining the associations between dietary fibers and diseases. We developed the Bio‐taxonomic Hierarchy Weighted Aggregation algorithm and generated a candidate list of dietary fibers with therapeutic potential. Notably, several of these fibers have been validated by previous studies, clinical trials, or our murine models, which underscores their relevance in potential therapeutic applications.

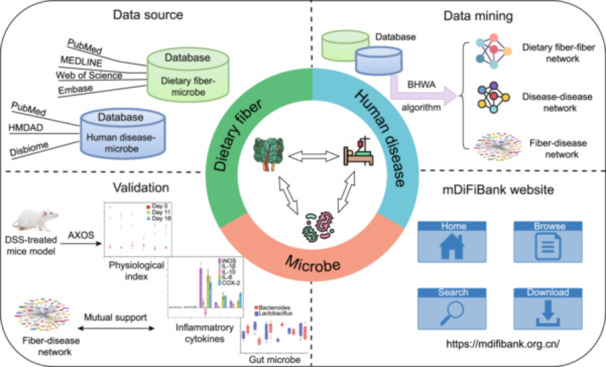

## INTRODUCTION

Microbiota inhabits various parts of the human body, such as the intestine, skin, oral cavity, nasal cavity, and reproductive organs. It coexists in a mutually beneficial relationship with the host and has undergone co‐evolution [1]. Numerous experiments have demonstrated that dysbiosis of gut microbiota is associated with inflammation [2], gut barrier impairment [3], metabolic disorders [4], and contributes to gastrointestinal inflammatory bowel diseases [5] (e.g., Crohn's disease, ulcerative colitis) and autoimmune diseases [6], as well as obesity [7, 8], cardiovascular diseases [9], type II diabetes [10, 11, 12], and cancers [13, 14].

Dietary fiber possesses key physicochemical properties like solubility, viscosity, and fermentability [15, 16]. Consuming fiber‐rich foods, fermented by gut microbiota to produce metabolites like short‐chain fatty acids [17, 18, 19], is an effective strategy to regulate gut microbiota and maintain health [20, 21]. Epidemiological and interventional studies affirm dietary fiber's significance in disease prevention, management, and mortality reduction [22, 23]. For instance, a systematic review of 1351 abstracts (selecting 18 meta‐analyses containing 298 prospective observational studies) showed that among the 21 evaluated health outcomes, 29% (six) were significantly improved, and dietary fiber intake was associated with reduced risks of cardiovascular diseases, atherosclerosis, and hypertension [24, 25, 26], pancreatic cancer, and gastric cancer [27]. Hence, ensuring an adequate intake of dietary fiber in a balanced diet is of utmost importance for gut health and reducing the risks of microbiota‐related diseases.

Despite the progress made in understanding the role of dietary fiber in health, research on the complex relationships among dietary fiber, disease, and the microbiota remains limited and fragmented. For example, some databases (Human Microbe‐Disease Association Database (HMDAD) [28], Disbiome database [29], MicroPhenoDB [30]) on microbe‐disease interactions have limitations, such as including non‐healthy controls, inconsistent identification systems, and insufficient utilization of multi‐dimensional microbial disturbances data. Other databases (NutriChem [31], FooDisNet [32]) that analyze diet‐related small molecules overlook specific dietary categories with a broad definition of diet and lack microbiome perspective insights. Notably, no existing database has been constructed to establish a network specifically for dietary fiber, nor has any categorization efforts focused on the functional aspects of dietary fiber in influencing microbial disturbance.

**Figure 1:**
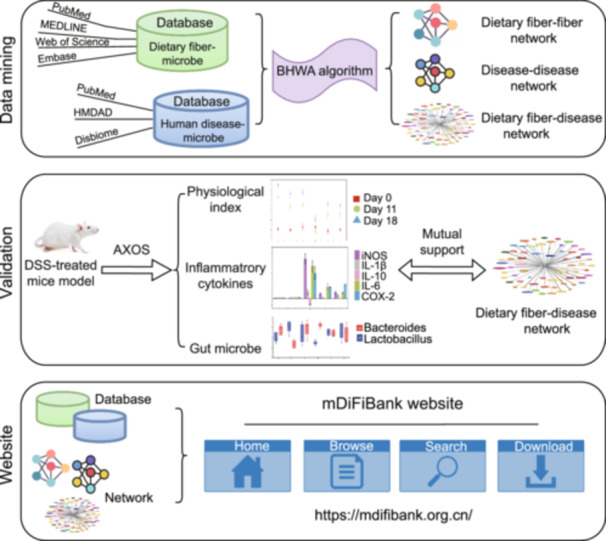
The study overview. The study included three primary components: Data mining, Validation, and mDiFiBank website. The “Data mining” component primarily introduced the data sources related to the dietary fiber‐microbe database and human disease‐microbe database, and involved the construction of microbiota‐based fiber–fiber network, disease‐disease network, and fiber‐disease network. This was achieved by using Bio‐taxonomic Hierarchy Weighted Aggregation (BHWA) algorithm to quantify the associations between the dietary‐fiber‐microbe database collection and the human disease‐microbe database collection. The “Validation” component utilized murine models of inflammatory bowel disease to validate the accuracy of the microbiota‐based dietary fiber‐human disease network relationship. The “Website” component encompassed the setup of the mDiFiBank website, offering four main functions: home, browse, search, and download.

This study used large‐scale text mining of 10,899 evidence from 1173 articles with integrative and comparative computational methods, Bio‐taxonomic Hierarchy Weighted Aggregation (BHWA) algorithm (Figure S1), to reveal relationships among dietary fiber, diseases, and microbiota, and understand functional similarities (Figure 1). We developed the mDiFiBank website (https://mdifibank.org.cn/) to explore network relationships, to query evidence, and to provide dietary recommendations based on microbial disturbance similarities for disease management and health improvement.

## RESULTS

### Developing a computational workflow for cumulative profiling of microbiota disturbances

Our initial step involved compiling a comprehensive data set of dietary fiber‐microbe interactions from major scientific databases, including PubMed, MEDLINE, Web of Science, and Embase. This process was guided by stringent screening criteria and de‐duplication procedures to ensure data accuracy and relevance (Figure S2). We prioritized randomized controlled trials (RCTs) as they are widely recognized as the gold standard for evaluating intervention efficacy. This focus aimed to minimize bias and provide robust evidence of the impacts of dietary fiber interventions. As a result, the data set comprised 317 evidence linking dietary fibers to microbiota changes, encompassing 54 types of dietary fibers and 112 human microbes extracted from 81 publications.

**Figure 2:**
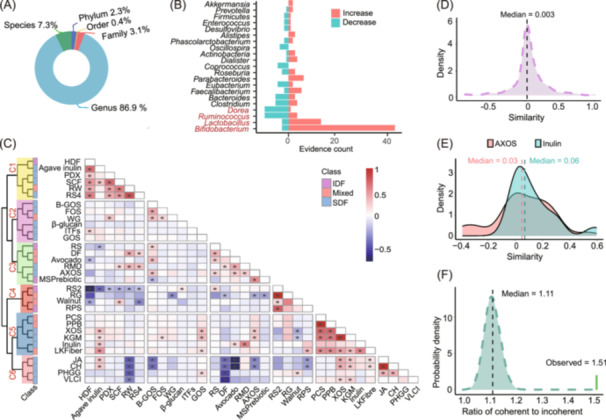
Dietary fiber‐microbe database construction and dietary fiber classification. (A) Microbial taxonomic distribution associated with dietary fiber. Five taxonomic levels (species to phylum) were filled in distinct colors. No microbe was identified at the class level. The proportions of evidence at each taxonomic level were shown relative to the total evidence in the dietary fiber‐microbe database. (B) Evidence counts supporting directional changes in the abundance of the top 21 microbes in response to dietary fiber intervention. Bar heights represented the number of publications indicating an increase or decrease in microbial abundance compared to a normal diet, with the *Y*‐axis showing the microbes associated with dietary fiber intervention. Dietary fiber types were color‐coded based on their solubility properties, with the color scheme matching that of the class legend. (C) Fiber‐fiber similarity based on microbiota disturbance comparison. The color gradient in the cells ranges from red to blue, indicating positive to negative similarities between dietary fibers. Significant similarities at a false discovery rate of 0.05 were marked with asterisks. Fiber groups C1–C6 were clustered based on microbiota disturbance similarities, distinguished by different colors. Fiber types were color‐coded based on their solubility properties, with the color scheme matching that of the class legend. Diagonal similarity values of 1 were omitted. Fiber categories include: soluble dietary fiber (SDF), insoluble dietary fiber (IDF), and Mixed (containing both SDF and IDF, or fibers with unclear definitions in the literature). Abbreviations for dietary fibers were used, with full names provided in Table S5. (D) The distribution of similarity between dietary fibers. The *X*‐axis represented pairwise dietary fiber similarities, and the *Y*‐axis showed the density of these similarities. The median value was derived from the distribution of similarities. (E) The similarity distributions of inulin and arabinoxylan oligosaccharides (AXOS). Different colors represented the similarity distributions of inulin and AXOS, with the *X*‐axis indicating the similarity of other fibers to inulin or AXOS and the *Y*‐axis representing the density of these similarities. The median values from these distributions were also presented. (F) The distribution density of three‐node loops in the dietary fiber network. The *X*‐axis represented the ratio of coherent to incoherent loops, while the *Y*‐axis showed the probability density of this ratio from a randomization test. The observed ratio value in the fiber–fiber network and the median ratio from the randomization test were indicated.

Among the dietary fibers, inulin—comprising a group of polysaccharides from specific tuberous plants—was notably prevalent, appearing in 11% of the publications (Figure S3A). In contrast, disturbances in *Bifidobacterium*, a common inhabitant of the gastrointestinal tract (GI tract), were reported in 78% of the literature (Figure S3B). The early stages of this research predominantly employed 16S rRNA sequencing, transitioning later to shotgun metagenomic sequencing. This technology resulted in a multi‐level data set where genus‐level data accounted for up to 87% of the microbial taxonomic distribution (Figure 2A).

To enhance the reliability of similarity assessments, we excluded evidence involving dietary fibers and microbes with fewer than three documented microbial disturbances. This refinement led to a data set of 261 evidence, encompassing 32 dietary fibers and 88 microbes. Analysis of this data set revealed trends in microbial responses to dietary fiber interventions, with some microbe' relative abundance increasing while others decreased (Figure 2B and Figure S3C). For example, 49 articles reported an increase in *Bifidobacterium* relative abundance, whereas only three indicated a decrease. Similarly, *Lactobacillus* abundance tended to increase, supported by 83% of the evidence set, while *Dorea* and *Ruminococcus* abundances generally decreased, as indicated by 92% and 73% of the evidence set, respectively.

To fully leverage the multi‐level information of microbiota data and better characterize the similarity of the research objects, we developed the BHWA algorithm for assessing the similarity between microbiota disturbances induced by dietary fiber or disease (Figure S1). Our algorithm workflow consisted of four steps, annotation, quantification, cosine similarity calculation, and weighted similarity determination, which could reflect the overall microbiota disturbance pattern. Our algorithm effectively utilized the multi‐taxonomy characteristics of microbial data to more accurately characterize the microbiota‐based similarity between any types of sources that may induce or be associated with the microbiota changes.

### Classifying dietary fibers by microbiota disturbance functionality

Dietary fibers are known to significantly impact microbiota disturbances, and our approach was to classify these fibers based on their effects on microbial patterns. We applied complete linkage hierarchical clustering to group the 32 dietary fibers into six categories (C1–C6) based on their microbiota disturbance patterns (Figure 2C). This method represented an advancement over traditional classifications, which typically divided fibers into solubility categories: soluble dietary fibers (SDF) and insoluble dietary fibers (IDF). Here, fibers that contained both SDF and IDF, or had no clear definition in the literature, were classified as “Mixed.” Our analysis revealed that categories C2, C5, and C6 contained only SDFs except mixed, which suggested that fibers with similar solubility tended to cluster and influence gut microbiota in distinctive ways.

In particular, we investigated resistant starch (RS) and its subtypes (RS1, RS2, RS3, RS4, and RS5). The analysis indicated that RS2 and RS4 had a negative similarity in microbial disturbance (−0.18, FDR = 0.5 × 10^−^^12^, Figure 2C), pointing to their opposite effects on microbiota. Additionally, RS2 exhibited high similarity with refined grains (RG) in terms of microbiota disturbance (0.99, FDR = 3.12 × 10^−^^13^, Figure 2C), suggesting that RS2 might mediate the microbiota disturbance induced by RG.

The distribution of pairwise similarities among dietary fibers largely followed a bell‐shaped curve with a median value of 0.003 (Figure 2D). Notably, arabinoxylan oligosaccharides (AXOS) and Inulin, two extensively studied fibers, showed distinct distributions of pairwise similarities. AXOS had a platykurtic and negatively skewed distribution with a median of 0.03, while inulin's distribution was leptokurtic with a median of 0.06 (Figure 2E), suggesting that AXOS acted differently while inulin acted similarly in modulating microbiota if compared to other fibers.

With pairwise fiber similarities computed, we constructed a network consisting of 32 dietary fibers (nodes) and 495 correlations (edges). This network featured three‐node loops, which are akin to network motifs and reflect network characteristics. We identified 4820 three‐node loops, representing 97.2% of the theoretical maximum (4960). Among these, 2901 were coherent, and 1919 were incoherent, resulting in a coherence‐to‐incoherent ratio of 1.51. Randomization tests (10,000 iterations) showed that the observed coherence was 1.34 times higher than expected by chance (Figure 2F), suggesting a significant tendency towards coherent three‐node loops and highlighting the prevalence of multilateral relationships among dietary fibers.

Finally, we visualized a network of 111 significant edges (FDR < 0.05), including 78 positive and 33 negative links among the 32 dietary fibers. This network was interactively accessible on our web tool (https://mdifibank.org.cn/), where users can explore and visualize the relationships between fibers. Positive microbial disturbance patterns were denoted by red lines, while blue lines indicated opposite patterns. Users can zoom into specific subnetworks by selecting any node of interest.

### Establishing between‐disease associations underpinned by similar microbiota

To investigate the relationships between diseases based on microbiota disturbances, we compiled a comprehensive disease‐microbe database from three primary sources: the HMDAD Database [28], the Disbiome database [29], and manually searched evidence from PubMed linking human diseases and microbes. We used microbiota profiles from healthy individuals as a baseline and recorded changes induced by diseases as microbiota disturbances. After processing (including removal of duplicates and mapping names based on disease etiology), we obtained 7779 evidence involving 161 disease groups (this defined the granularity of the term “disease” in this study) and 1066 microbes for further analysis.

**Figure 3:**
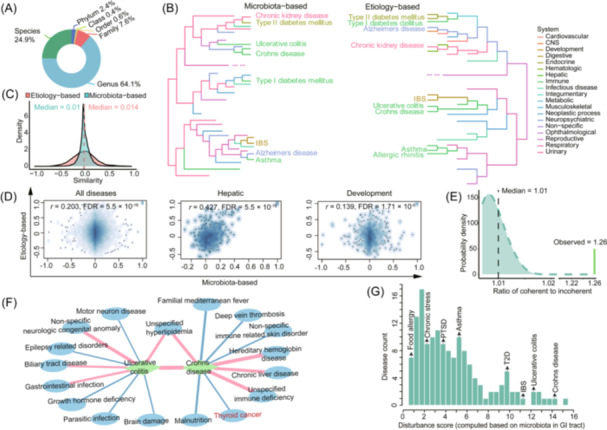
Disease‐microbe database construction and associations between diseases. (A) Composition of taxonomy‐specific microbe‐disease associations. The composition of the six taxonomic levels (species to phylum) was represented using distinct colors. The values indicated the proportion of evidence at each level relative to the total evidence in the disease‐microbe database. (B) Phylogenetic tree view of disease clusters based on microbiota disturbance data (left) and etiology information data (right). Diseases and branches were colored by general disease categories, as shown in the legend. The figure selectively highlighted certain diseases while omitting those belonging to other branches. (C) The distributions of microbiota‐based and etiology‐based pairwise disease similarities. Different colors represented the distributions of these similarities, with the *X*‐axis showing pairwise disease similarities and the *Y*‐axis indicating similarity density. Median values from these distributions were also presented. (D) Correlation between microbiota‐based and etiology‐based disease similarity. The first panel showed the correlation across all 161 disease groups, while the second and third panels focused on correlations within the hepatic and developmental systems, respectively. (E) The distribution density of coherent to incoherent loops in disease network. The *X*‐axis represented the ratio of coherent to incoherent loops, while the *Y*‐axis showed the probability density of this ratio from a randomization test. The observed ratio in the disease‐disease network and the median ratio from the randomization test were indicated. (F) Crohn's disease‐ and ulcerative colitis‐centric association network. A subnetwork was constructed, consisting of the top five diseases exhibiting consistent microbial disturbance directions and the top five diseases showing opposite microbial disturbance directions compared to Crohn's disease and ulcerative colitis. Blue edges denoted negative similarity between disease pairs, while red edges indicated positive similarity. (G) Distribution of diseases' disturbance scores specific to gastrointestinal (GI) tract. The *X*‐axis showed the range of disturbance scores computed from microbiota data in the GI tract. Bar heights represented the number of diseases within each score interval, with specific disease names listed above the bars indicating corresponding disturbance score ranges. CNS, central nervous system; IBS, irritable bowel syndrome; PTSD, posttraumatic stress disorder; T2D, type II diabetes.

Analysis showed that Crohn's disease is the most extensively studied condition, with 52 evidence (Figure S4A), and *Prevotella* as a key microbe associated with various diseases (222 evidence, Figure S4B). The microbial data were categorized across six hierarchical levels, with the genus level representing the largest proportion, at 64%, followed by the species level (Figure 3A). Most microbial taxa exhibited both increases and decreases depending on the disease state, but some microbes, such as *Faecalibacterium* and *Shigella*, showed predominant trends of decrease and increase, respectively, across different diseases (Figure S4C). For example, *Prevotella* increased in nonspecific gingival periodontal diseases and female reproductive organ diseases but decreased in asthma and Parkinson's disease (Figure S4D).

Diseases with similar microbiota disturbance profiles were expected to be associated with each other. Using our BHWA algorithm, we quantified microbiota disturbances across disease pairs (microbiota‐based similarity) and conducted hierarchical clustering of the 161 diseases based on microbiota similarity (Figure 3B, Figure S5A). We also performed clustering based on etiology (Figure 3B, Figure S5B) using high‐dimensional space representation derived from electronic diagnosis records of over 151 million Americans, embedding the disease's etiological information into space coordinates [33, 34]. Interestingly, the two clustering approaches revealed different groupings for several diseases: asthma and Alzheimer's disease were close in microbiota‐based clustering but distant in etiology‐based clustering; although irritable bowel syndrome and inflammatory bowel disease (ulcerative colitis and Crohn's disease) often exhibit similar symptoms, complicating the diagnosis process, they can be discerned in this study by showing distinct microbiota disturbance; type II diabetes and kidney diseases were grouped together in microbiota‐based clustering, offering a microbial explanation for their high co‐occurrence in clinical practice.

We systematically compared two types of similarity measures, computed based on microbial disturbance and diagnosis records, respectively. On average, etiology‐based similarity between diseases tended to be stronger than the corresponding microbiota‐based similarity (Figure S6). One possible reason was that microbial disturbances capture the underlying variations in the composition and activity of the microbiota, which can be influenced by mixed factors such as diet, environment, and host genetics. However, etiology‐based calculation mainly focused on observable clinical characteristics in diagnosis records that are more directly associated with disease manifestations. The similarity distribution for etiology‐based measures was more dispersed, while that for microbiota‐based measures was more concentrated (Figure 3C). The difference between etiology‐based and microbiota‐based similarities was bell‐shaped, with a median of −0.005 (Figure S4E). Pearson's correlation analysis confirmed a significant concordance between the two types of similarity measures, with a correlation coefficient of 0.2 (FDR = 5.5 × 10^−^^16^; Figure 3D, Figure S7). This consistency varied across disease categories, with the highest correlation in hepatic diseases (0.43, FDR = 5.5 × 10^−^^16^) and the lowest in developmental diseases (0.14, FDR = 1.71 × 10^−^^16^; Figure S7).

We constructed a disease network where diseases with similar microbiota disturbances were more closely connected. This network included 161 diseases (nodes), 12,850 disease‐microbe correlations (edges), and 678,001 three‐node loops, representing 99.3% of theoretical values (682,640). Among these loops, 377,534 were coherent, and 300,467 were incoherent, resulting in a coherence‐to‐incoherence ratio of 1.26. Randomization tests (10,000 iterations) showed that the observed values for coherent loops were 1.23 times higher than expected by chance, indicating a tendency for coherence within the disease network (Figure 3E). This highlighted the complexity of disease relationships based on microbiota disturbances.

For enhanced visualization, we filtered the disease similarity network to include only significant associations with an FDR < 0.05. The filtered network included 4877 relationships, with 3192 positive links and 1685 negative links among the 161 diseases. This network was available on our website (https://mdifibank.org.cn/), where users can explore disease relationships interactively. Red edges represented positive microbial similarity, and blue edges denoted negative microbial similarity. For example, we illustrated the analysis with Crohn's disease and ulcerative colitis, showing a positive similarity of 0.46 (FDR < 10^−16^) indicating similar microbial disturbance patterns (Figure 3F). In contrast, thyroid cancer and Crohn's disease exhibited a negative similarity of −0.16 (FDR = 7.75 × 10^−5^), suggesting opposite‐direction changes in microbiota (Figure 3F).

Considering variations in microbial composition across body sites, we performed separate analyses for different body regions. We identified 39 distinct body sites and generated a multi‐level body‐site‐specific information matrix. We measured the deviation between disease and health states by the Euclidean distance between two vectors, defining it as the body‐site‐specific disturbance score (“*DS*”) of the disease. We have provided the list of the *DSs* (Table S1) as well as the statistical summary (Table S2). From the top five ranked sample sources, it can be observed that the ability of microbial disturbances induced by the same disease varies across different body sites (Figure S8A). For instance, in the GI tract, the microbial *DSs* range from 0.62 to 15.1 (Figure 3G). A higher score indicated a greater capacity of this disease category to disturb the microbiota in the GI tract. Among them, diseases like type II diabetes (*DS* = 9.95), ulcerative colitis (*DS* = 12.01), and Crohn's disease (*DS* = 14.01) exhibited higher disturbance capacities compared to food allergy (*DS* = 0.74), likely due to their substantial effects on immune response, inflammation, and tissue damage.

### Linking dietary fiber to disease consequences via microbiota disturbance

**Figure 4:**
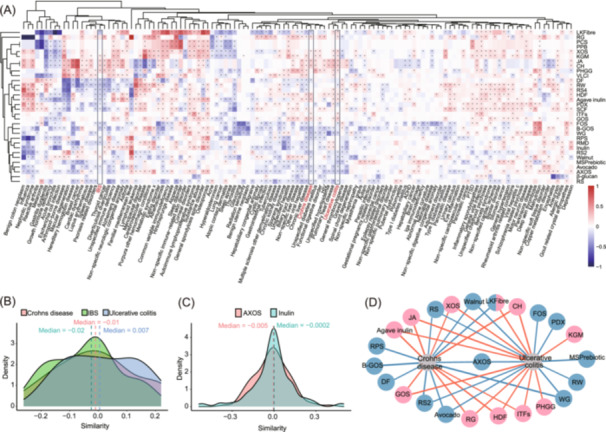
Dietary fiber‐disease associations linked by microbiota disturbance. (A) Heatmap of fiber‐disease associations based on microbiota disturbance comparison. It examined the interactions between 32 dietary fibers and 123 human diseases. The *X*‐axis and *Y*‐axis displayed disease systems and dietary fiber classifications, respectively. Cells were color‐coded from red to blue to represent positive to negative associations between dietary fibers and diseases. Significant associations at a false discovery rate of 0.05 were marked with asterisks. (B) The similarity distributions of fibers' associations with Crohn's disease, IBS, or ulcerative colitis. Different colors represented the similarity distributions of fiber associations with these diseases. The *X*‐axis indicated the similarity of a fiber's association with a disease, while the *Y*‐axis showed the density of this similarity. Median values were highlighted. (C) The distributions of diseases' associations with inulin or AXOS. This panel showed how diseases' associations with these dietary fibers are distributed. Different colors indicated the distributions, with the *X*‐axis representing the similarity of diseases' association with a fiber and the *Y*‐axis representing similarity density. Median values were also shown. (D) Dietary fibers with significant positive and negative associations with Crohn's disease and ulcerative colitis. Blue links denoted negative associations, while red links indicated positive ones. Dietary fibers were shown with blue, red, or mixed colors to represent their association types with the diseases. Abbreviations for dietary fibers were used, with full names provided in Table S5.

Dietary fiber, a key component of daily nutrition, plays a vital role in modulating the gut microbiota as well as its interaction with the host [35]; the microbiota can then influence disease occurrence and development through various routes, such as the gut‐brain and gut‐liver axes [36, 37]. Using the BHWA algorithm, we computed microbiota disturbance similarities between dietary fiber and disease across various taxonomic levels. We identified 1659 significant fiber‐disease associations involving 32 dietary fibers and 123 diseases (FDR < 0.05, Figure 4A). The magnitude and sign of the microbiota‐based similarity reflected the strength and direction of the fiber‐disease association, respectively. The median similarity value of 0.001 suggested a trend where improper fiber intake slightly increased disease risks (Figure S9A). We classified 123 diseases into 19 systems according to the International Classification of Diseases, 9th Revision (ICD‐9). In our analysis, we found a significant positive relationship between refined grains (RG) and hepatic diseases based on the microbial disturbance patterns (Figure S9B). This suggested that consumption of RG might exacerbate pro‐inflammatory effects, whereas whole grains (WG) appeared to have an anti‐inflammatory effect. This finding was consistent with recent studies [38], which demonstrated that whole grain and fiber intake had a protective effect against chronic liver diseases. Similarly, it could be seen that β‐glucan and cardiovascular diseases had a negative relationship (Figure S9B). This suggested that consumption of β‐glucan also had a certain inhibitory effect on cardiovascular diseases and could alleviate inflammation, etc. This conclusion was also consistent with the results of previous studies [39, 40].

Crohn's disease and ulcerative colitis showed similar fiber associations, while IBS exhibited distinct patterns, reflecting their different disease profiles (Figure 4A). For the fiber distribution profiles, Crohn's disease and ulcerative colitis had a similar tendency, whereas irritable bowel syndrome showed a leftward skew (Figure 4B), indicating a greater number of dietary fibers with microbial disturbance patterns opposite to irritable bowel syndrome. Inulin and AXOS, the most extensively studied fibers, exhibited similar distribution patterns, indicating comparable effects in terms of mitigating or enhancing inflammation that led to disease onsets (Figure 4C).

Distinct fibers exert differential impacts on the same diseases. Inulin‐type fructans (e.g., Jerusalem artichoke inulin (JA), Chicory inulin, Agave inulin), showed disturbance patterns akin to Crohn's disease and ulcerative colitis (Figure 4D), implying a potential exacerbatory inflammation. Previous studies have demonstrated that dietary inulin triggers microbiota‐derived bile acids and promotes type 2 inflammation at mucosal surfaces. Specifically, inulin supplementation elevates bile acid levels produced by gut microbiota, which in turn activates immune cells to produce the inflammatory cytokine IL‐5, thereby contributing to intestinal inflammation and worsening inflammatory bowel disease [41, 42]. The results were consistent with our experiment and helped us prove the accuracy of the model's predictions. Conversely, AXOS, walnuts, and whole grains exhibited patterns opposite to these diseases (Figure 4D), potentially alleviating inflammatory responses. Such a differential effect highlights the significance of fiber type in regulating gut health and inflammation.

Our webtool (https://mdifibank.org.cn/) provided interactive visualizations of these relationships. Users could explore how dietary fibers influence diseases, view ranked associations, and identify key disturbed microbes. Blue edges indicated fibers that might mitigate disease‐related microbiota disturbances, while red edges suggested fibers that could worsen them (Figure 4D).

### Validating preventive and therapeutic effects of arabinoxylan on murine models with inflammatory bowel disease

**Figure 5:**
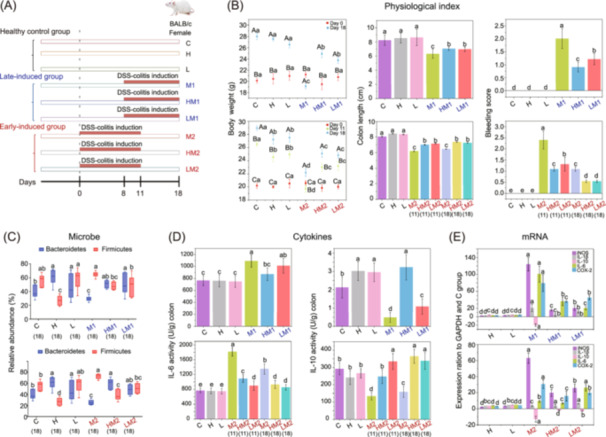
Preventive and therapeutic effects of arabinoxylan on murine models of inflammatory bowel disease. (A) Experimental design for studying the effects of arabinoxylan in murine models of inflammatory bowel disease. Female BALB/c mice were treated with dextran sulfate sodium (DSS) to induce colitis and administered arabinoxylan orally at either low (0.2 g/kg) or high (0.4 g/kg) doses. Mice were divided into three types with nine groups. Healthy control group: Group 1 (control, C) received normal water, while Group 2 (high dose arabinoxylan, H) and Group 3 (low dose arabinoxylan, L) were given arabinoxylan at high and low doses until day 18. Groups 4 to 6 (late‐induced group) received arabinoxylan until day 18 and induced DSS from days 8 to 18. Groups 7 to 9 (early‐induced group) received arabinoxylan until day 18 and induced DSS from days 1 to 11. (B) Comparison of mice physiological index between control and late‐induced (upper) and early‐induced groups (lower). Body weight, colon length, and bleeding score were measured on different days. Red, green, and blue dots with error bars represented body weight measurements on days 0, 11, and 18. Bar heights with error bars showed colon lengths and bleeding scores for various groups. Capital letters represented the differences of the same group under different days, while lowercase letters represented the differences between different groups on the same day. The numbers in parentheses denoted the day of measurement. (C) The relative abundance of microbes at the phylum level between control and late‐induced (upper) and early‐induced groups (lower) on day 18. The *X*‐axis represented different mouse groups, while the *Y*‐axis showed the relative abundance of disturbed microbes due to arabinoxylan intervention. Different microbial abundances were color‐coded with error bar (as shown in legend). (D) Cytokines secretion comparison between control and late‐induced (upper) and early‐induced groups (lower). The *X*‐axis represented different mouse groups, while the *Y*‐axis showed interleukin 6 (IL‐6) and interleukin 10 (IL‐10) levels. Bar heights and error bars indicated the secretion amount of these cytokines in various groups. (E) Gene expression changes of five inflammatory cytokines in colon tissue from late‐induced (upper) and early‐induced groups (lower). The *X*‐axis represented different mouse groups (healthy control, late‐induced, and early‐induced groups), and the *Y*‐axis showed relative mRNA expression levels. Bars with different colors represented specific inflammatory cytokines, as shown in legend. Letter “a,” “b,” and “c” and others above the bars denoted statistical significance between groups, with identical letters indicating no significant difference and different letters indicating significant differences. The numbers in parentheses denoted the day of measurement. COX‐2, cyclooxygenase‐2; iNOS, inducible nitric oxide synthase; IL‐1β, interleukin‐1 beta.

To validate the accuracy and reliability of the inferred dietary fiber‐disease network, we conducted experiments to verify the relationships between several key nodes within the network. Specifically, we employed murine models with dextran sulfate sodium (DSS)‐induced colitis to assess the impact of arabinoxylan (its hydrolysis product was AXOS [43, 44]) on inflammatory bowel disease. Our study involved mice categorized into the healthy control group (labeled as C, H, and L), the late‐induced inflammation group (for investigating the preventive effect of arabinoxylan; labeled as M1, HM1, and LM1), and the early‐induced inflammation group (for examining the therapeutic effect of arabinoxylan; labeled as M2, HM2, and LM2). Each of these groups was further subdivided into three subgroups: basal diet, high‐dose arabinoxylan feed (labeled with the prefix “H”), and low‐dose arabinoxylan feed subgroups (labeled with the prefix “L”; Figure 5A).

First, we measured three physiological indices of colitis severity in murine models: body weight, colon length, and bleeding score (Figure 5B). Compared to the healthy group (C), mice with colitis, whether induced late or early (M1 or M2), exhibited significant decreases in body weight and colon length, as well as increased fecal bleeding, indicative of typical symptoms of colonic inflammation. However, these colitis‐induced changes became less pronounced in mice fed with arabinoxylan. Specifically, we compared the high‐dose arabinoxylan groups: HM1 versus M1, or HM2 versus M2, and the low‐dose groups: LM1 versus M1 or LM2 versus M2 on day 11 and day 18. In these comparisons, we used repeated measures analysis of variance, and the *p*‐values were all less than 0.01 (Figure 5B). These findings underscored the potential of arabinoxylan (or AXOS) in ameliorating inflammatory bowel disease.

To elucidate the mechanism underlying arabinoxylan's alleviation of inflammatory bowel disease, we conducted further investigations into microbial disturbance, cytokine secretion, and gene expression.

Microbiota composition analysis revealed that, compared to the control group C, mice with colitis induced late and early (M1 and M2) showed decreased relative abundance of *Bacteroidetes* phylum while increased relative abundance of *Firmicutes* phylum (Figure 5C). Arabinoxylan intervention in all early‐induced groups (HM2 and LM2) could reverse the altered relative abundance of *Bacteroidetes* phylum and *Firmicutes* phylum induced by colitis (M2 group). This reversal effect was dose‐dependent, i.e., higher dose of arabinoxylan showed greater efficacy in modulating the abundance of these bacteria (Figure 5C). Arabinoxylan intervention in all late‐induced groups (HM1 and LM1) could also reverse the altered relative abundance of *Bacteroidetes* phylum and *Firmicutes* phylum induced by colitis (M1 group), although the reversal effects were less pronounced compared to the early‐induced groups (Figure 5C). In addition, compared to the control group C, mice with colitis (M1) exhibited increased relative abundance of *Bacteroides*, while mice with colitis (M2) exhibited increased relative abundance of *Bacteroides* and decreased relative abundance of *Lactobacillus* at the genus level (Figure S10A). Arabinoxylan intervention in all late‐induced (preventive effect) groups and early‐induced (therapeutic effect) groups can also reverse the altered relative abundance of *Bacteroides* and *Lactobacillus* genus induced by colitis (Figure S10A). In summary, the disturbance pattern of *Bacteroidetes* and *Firmicutes* phylum, as well as *Lactobacillus* and *Bacteroides* genus in response to arabinoxylan was opposite to the disturbance pattern in response to inflammatory bowel disease, which was consistent with the findings reported in the preceding sections of the results.

Cytokines, pivotal in intestinal mucosa inflammation, were assessed using ELISA. Compared to the control group C, interleukin 6 (IL‐6, Figure 5D), inducible nitric oxide synthase (iNOS, Figure S10B), cyclooxygenase‐2 (COX‐2, Figure S10B), and interleukin‐1 beta (IL‐1β, Figure S10B), exhibited elevated activity levels, while interleukin 10 (IL‐10, Figure 5D) showed reduced activity level in mice with colitis induced late and early (M1 vs. C and M2 vs. C; *p*‐values < 0.01). These colitis‐induced changes could be reversed after arabinoxylan administration (HM1, LM1, HM2, and LM2 groups), and this effect was correlated with dose. Additionally, gene expressions of these cytokines aligned with the ELISA‐based observations (Figure 5E).

Overall, these experimental results demonstrated that arabinoxylan effectively reduced the expression of inflammatory cytokines in mice with inflammatory bowel disease and restored gut microbiota disturbance induced by the disease. This aligned with our network predictions, showcasing the reliability of the associations between dietary fibers and diseases in our study and the feasibility of utilizing our BHWA algorithm to compute microbial disturbance patterns.

## DISCUSSION

Epidemiological and interventional studies underscore the significant impact of dietary fiber on disease development, management, and mortality [15, 23, 26]. However, systematic approaches for providing personalized dietary guidance are lacking. With the growing availability of biomedical data, bioinformatics tools enable predictive analysis of dietary fiber‐disease associations, offering insights into the complex interplay between diet, microbiota, and disease [45, 46].

Unlike previous studies that focused on small molecular components, proteins, and pathways to infer dietary‐disease associations [31, 32, 47], our approach delves into microbiota disturbances. Various techniques, including 16S amplicon sequencing, microarrays, and metagenomics, provide insights at different taxonomic levels [48, 49, 50, 51, 52]. Traditional similarity calculations often focus on specific taxonomic levels [28], limiting data utilization. Our study introduced the BHWA algorithm, which integrates microbial data across all taxonomic levels using inverse‐variance weighting. This method enhanced microbial data utilization and enabled a nuanced understanding of similarity relationships between dietary fibers and diseases. Using BHWA algorithm, we constructed fiber‐fiber, disease‐disease, and fiber‐disease networks, demonstrating the intricate linkages between dietary fibers and human maladies by sharing similar microbiota disturbance patterns.

In the fiber–fiber network, greater disturbance similarity among different dietary fibers indicated a higher degree of functional similarity among them. Dividing the 32 dietary fibers into six functional categories can provide insight into the role and significance of dietary fibers. For some individuals sensitive to new strains, dietary fiber may offer a safer regulatory mode. This is because the strains introduced by fermented foods might provoke allergic reactions or intestinal discomfort in certain people. In contrast, dietary fiber can promote the growth of beneficial bacteria in the gut, potentially mitigating this risk. Some high‐sugar fruits, such as bananas and grapes, though rich in other nutrients, may cause rapid blood glucose spikes. Whole grains and insoluble cereal fibers are typically not viscous, do not significantly influence postprandial glucose responses (i.e., glycemic index), and are strongly fermented by the gut microbiota in the colon. Additionally, certain soluble fibers, such as β‐glucan found in oats, can bind to cholesterol in the intestine, reducing its absorption and ultimately lowering blood cholesterol levels [39]. This is one of the motivations for us to explore the functions of dietary fiber.

In the disease‐disease network, higher disturbance similarity among diseases indicated a greater likelihood of comorbidity or shared pathophysiology, considering that the microbiota disturbance can serve as the risk factor or mediator shared by comorbid diseases. For instance, asthma and type II diabetes frequently coexist, demonstrating an association but lacking an explaining mechanism [53, 54]. A single taxonomic level was inadequate to characterize their similarity, as evidenced by a negative similarity at the genus level (−0.04, *p* = 0.38). However, a significant microbiota disturbance similarity of 0.06 (*p* = 0.03) was identified using multi‐level microbiota data with the BHWA algorithm, suggesting a potential microbial mechanism for their coexistence (Figure S8B and Table S3). For another example, ulcerative colitis and Crohn's disease, both classified as inflammatory bowel diseases [55, 56], showed a significant microbial disturbance similarity of 0.46 (*p* = 1.54 × 10^−29^), contrasting with the negative similarity (−0.16, *p* = 2.98 × 10^−8^) observed in growth hormone deficiency, a disease from a different system (Figure S8C and Table S4). This suggested that microbial disturbance patterns can aid in disease classification.

In the fiber‐disease network, we hypothesized that dietary fiber induces opposite microbial disturbance patterns compared to those linked to disease, suggesting their potential benefits for improving disease states. The network included 123 diseases and 32 dietary fibers, offering 1659 dietary recommendations for specific patients. For example, increasing β‐glucan intake lowers colorectal cancer risk and reduces inflammation, supporting previous findings that it can decrease mortality and cell apoptosis in colorectal cancer patients [57, 58]. This approach to dietary recommendations parallels gene expression‐based drug repurposing, where a drug is considered effective if it can reverse disease‐associated gene expression patterns [59, 60]. However, our approach, focusing on microbiota disturbances, provided valuable dietary recommendations but requires further validation in clinical settings. Individual variability, nutritional factors, intake dosages, and multifactorial pathogenesis can influence outcomes. Translating these findings into practice necessitates a personalized approach considering each patient's condition.

Additionally, our data rely on published research, which may have limitations such as publication bias and incomplete results. Ongoing efforts are needed to update and expand the database, incorporating new evidence to enhance dietary guidance for a broader patient population. During the data collection process, since most of the datasets relied on were not publicly available, we were unable to quantify the exact magnitude of microbiota changes. Therefore, we only considered the direction of microbial disturbance rather than the intensity of the change. While we recognize that this is a limitation, we hope that our study can serve as a foundation for future research. We are optimistic that with the availability of more granular strain‐level data in the future, it will be possible to incorporate the intensity of microbiota changes into subsequent studies, enhancing the precision of our model.

## CONCLUSION

This investigation explored the connections between dietary fibers and diseases through the lens of microbial disturbances. By analyzing the similarities in microbiota disturbance pattern with a robust framework for BHWA algorithm, we developed scientifically grounded dietary recommendations tailored to specific disease subpopulations. Our findings provided valuable insights for personalized dietary interventions aimed at managing or preventing diseases, thereby offering a theoretical framework for improving health outcomes through targeted dietary guidance.

## METHODS

### RCTs evidence of causality between dietary fiber and microbial abundance change

We conducted a comprehensive search for published articles on the relationship between dietary fiber and human microbiota using relevant keywords that adhered to the PICOS principle, as illustrated in Figure S2. Our search included databases such as Embase (https://www.embase.com/), PubMed (https://pubmed.ncbi.nlm.nih.gov/), MEDLINE (https://www.nlm.nih.gov/medline/), and Web of Science (https://www.webofscience.com/), with a cutoff date of February 2023. We manually reviewed project information to verify the relevance of the articles, focusing on those addressing dietary fiber's impact on human microbiota based on abstracts and results. The selected studies were then consolidated and de‐duplicated before full‐text screening.

We applied specific criteria to ensure the quality and reliability of the selected studies. Eligible studies included RCTs, cluster RCTs, or quasi‐RCTs, as these designs were considered the gold standard for assessing intervention effectiveness due to their ability to minimize bias and provide robust evidence. The studies had to involve healthy participants and interventions aimed at increasing dietary fiber intake. The term “healthy participants” referred to those with all normal physiological indicators, excluding special period populations such as infants, pregnant women, and those in pregnancy and lactation. The inclusion criteria for participants were as follows: (1) free of metabolic and gastrointestinal diseases, with no history of such diseases, (2) avoiding medications known to affect intestinal function, (3) free of antibiotic use for at least the past 8 weeks, (4) limiting alcohol consumption to 2 servings/d (e.g., <28 g ethanol/d), (5) avoiding taking prebiotics or probiotics; (6) consuming a moderate fiber diet, (7) continuing to consume the same dose of vitamin and/or mineral supplements, if applicable, (8) maintaining the current level of physical activity, and (9) agreeing to keep detailed dietary and stool records. Additionally, comparator groups for food interventions needed to include a placebo or habitual diet group. The primary outcome of interest was the change in microbial composition at the end of the intervention period.

Our final dietary fiber‐microbe database comprised 317 evidence from 81 publications, covering 54 dietary fibers and 112 human microbes. For clarity, dietary fiber abbreviations, sources, and detailed descriptions are listed in Table S5. To ensure data set reliability, we excluded evidence of dietary fibers and microbes with fewer than three types of microbial disturbances based on the frequency distribution of corresponding microbes for each dietary fiber (Figure S3D). This resulted in a refined data set of 261 evidence involving 32 dietary fibers and 88 microbes for subsequent analysis.

### Curated database associating microbial abundance change with disease

To identify datasets linking microbiota disturbance with human diseases, we searched public sources, including Human Microbe‐Disease Association Database (HMDAD) [28] (https://www.cuilab.cn/hmdad), Disbiome [29] database (https://disbiome.ugent.be/home), and PubMed. HMDAD, a pioneer in exploring microbe‐disease associations, included data up to July 2014. For this study, we extended the search to include articles published from July 2014 to February 2023 using the PubMed database. We integrated and de‐duplicated association data from HMDAD and Disbiome, along with manually curated data from PubMed.

To ensure data quality and reliability, we focused on patients with a certain disease, with healthy participants serving as comparators. We focused on studies involving patients with specific diseases, using healthy participants as comparators. We excluded studies where participants were undergoing drug treatment, antibiotic intake, lactation, or surgical interventions to ensure that observed microbial changes were specifically related to the disease being investigated. Our final data set comprised 10,582 evidence involving 325 diseases and 1766 microbes from 1092 research articles.

Given that disease names varied across different projects, we standardized these names according to the International Classification of Diseases, 9th Revision (ICD‐9) and mapped them to etiology‐based disease groups using a disease embedding method. The ICD‐9 coding system was a standardized framework used worldwide to classify and code diagnoses, symptoms, and procedures. It allowed for the systematic grouping of diseases based on a combination of clinical and etiological characteristics, facilitating more precise clustering in our analysis. Furthermore, we ensured the standardization of disease names by adhering to these coding schemes, thus ensuring consistency and accuracy in disease classification across our data set.

The etiology‐based clustering was derived from mapping diseases to the ICD‐9, which groups diseases into 567 major diagnostic categories. These categories were determined using high‐dimensional space representations derived from electronic diagnostic records of over 151 million Americans. This approach embedded etiological information about diseases into spatial coordinates, facilitating the grouping of diseases according to their underlying causes [34]. The diseases were grouped based on clinically relevant manifestations, allowing us to categorize diseases in a way that reflects their etiology. In our analysis, we identified 161 overlapping disease types that were common to both the microbiota‐based similarity and the etiology‐based clustering (Table S6).

We consolidated the evidence on microbial disturbances for the same disease and filtered out evidence from diseases with fewer than three types of microbial disturbances. This process yielded a refined data set of 7779 evidence, encompassing 161 disease groups and 1066 microbes for further analysis.

### Bio‐taxonomic hierarchy weighted aggregation (BHWA) algorithm for measuring microbiota disturbance similarity

To accurately measure microbiota disturbance similarity between dietary fibers and diseases, we built upon methodologies by Ma et al. [28] and Yao et al. [30] and developed the BHWA algorithm. This approach involves four main steps: annotation, quantification, cosine similarity calculation, and weighted similarity determination (Figure S1).

Annotation: To ensure the authenticity and enhance the utility of this data, we annotated microbial taxa at multiple levels, including species, genus, family, order, class, and phylum. We utilized the SILVA138.1 database [61] (https://www.arb-silva.de) and the Greengenes [62] database (https://ngdc.cncb.ac.cn/databasecommons/database/id/3120) for this purpose. Both of these databases are widely regarded as reliable resources for microbial classification. Specifically, we downloaded the annotation information from Silva and Greengenes with the taxonomic hierarchies available, and then mapped the microbes (e.g., species‐ and genus‐level data) from our collected microbiota evidence sets to these annotations to infer higher taxonomic levels, such as family, order, class, and phylum. This approach allowed us to link lower levels of taxonomic classification (e.g., species or genus) to higher levels (e.g., family or phylum) when more specific data was not available. By inferring information from lower to higher taxonomic levels, we constructed multi‐level microbiota matrix data based on the raw evidence.

Quantification: For each level, we adapted the Equation (1) to calculate the quantitative strength of the disturbance evidence $D_{m,i}$ and the microbiota‐based fiber/disease cosine similarity ${\mathrm{SIM}}_{\mathrm{ij}}$. Taking the species level as an example, the following formula was demonstrated:

(1) $$D_{m,i}^{S}=\alpha_{m,i}^{S}\times C_{m,i}^{S}\times \ln(N^{S}/n_{i}^{S}).$$

Here the superscripts “S” appearing in the formula represented species level.

In the Equation (1), $\alpha_{m,i}^{S}\in \left\{\begin{array}{l} 1\\ -1 \end{array}\right.$ denoted the change direction of microbe m in relation to fiber or disease i. Specifically, $\alpha_{m,i}^{S}=1$ indicated an increase in microbe m for fiber or disease i, while $\alpha_{m,i}^{S}=-1$ signified a decrease in microbe m for fiber/disease i. The variable $C_{m,i}^{S}$ represented evidence count of reporting an association between a microbe m and a specific fiber i or disease i. To assign weights to microbes, we utilized the coefficient $\ln(N^{S}/n_{i}^{S})$ which can effectively reduce the weights of microbes that are globally associated with various fibers (or diseases) and increase the weights of microbes that are specifically associated with a few fibers (or diseases), which is designed to increase the discrimination and specificity of different fibers (or diseases). This weight coefficient affects the weight of fiber (or disease) similarity. It cannot change a non‐correlated fiber (or disease) pair to a correlated fiber (or disease) pair, and vice versa. Here, $N^{S}$ denoted the total number of fibers or diseases i at this taxonomic level (species), and $n_{i}^{S}$ represented the number of fibers or diseases i associated with microbe m.

Cosine similarity calculation: For species level, each dietary fiber or disease i can be represented by a vector $\vec{D_{i}^{S}}$ in the Equation (2), which contains the quantitative strength of disturbance evidence with microbes 1 to M.

(2) $$\vec{D_{i}^{S}}=\left\{D_{1,i}^{S},\cdot \cdot \cdot {,\;D}_{m,i}^{S},\;\cdot \cdot \cdot ,\;D_{M^{S},i}^{S}\right\}.$$

The cosine value was employed to calculate the similarity between the vectors $\vec{D_{i}^{S}}$ and $\vec{D_{j}^{S}}$ in Equation (3), providing a measure of similarity ${\mathrm{SIM}}_{i,j}^{S}$ between the microbial associations of pairwise fibers or diseases.

(3) $${\mathrm{SIM}}_{i,j}^{S}=\cos\left(\theta \right)=\frac{\sum_{m=1}^{M^{S}}D_{m,i}^{S}D_{m,j}^{S}}{\sqrt{\sum_{m=1}^{M^{S}}{\left(D_{m,i}^{S}\right)}^{2}}\;\sqrt{\sum_{m=1}^{M^{S}}{\left(D_{m,j}^{S}\right)}^{2}}}.$$

By comparing the cosine similarity values, we could assess the similarity or dissimilarity between different dietary fibers or diseases based on their microbial associations. To determine the significance of the similarity between the vectors $\vec{D_{i}^{S}}$ and $\vec{D_{j}^{S}}$, we calculated the *t*‐statistics using *p*‐value through the application of the unpaired two‐tailed Student's *t*‐test. Subsequently, the variance $\sigma_{i,j}^{S}$ could be obtained by the quotient of the cosine similarity ${\mathrm{SIM}}_{i,j}^{S}$ and the corresponding *t*‐statistics. The weight $W_{i,j}^{S}$ of each fiber or disease at species level was the inverse variance.

(4) $$\sigma_{i,j}^{S}=\frac{{\mathrm{SIM}}_{i,j}^{S}}{t_{i,j}^{S}},$$

(5) $$W_{i,j}^{S}=\frac{1}{\sigma_{i,j}^{S}}.$$

The aforementioned Equations (1)–(5) were equally applicable to similarity calculations for other hierarchical levels. Thus, after one round of computation, we could obtain the similarities at six levels and their respective level weights.

Weighted Similarity Determination: The weighted similarity ${\mathrm{SIM}}_{i,j}$ was obtained by combining the similarity at each taxonomic level with the corresponding weight value. This calculation was performed using the Equation (6) mentioned in the study. The weighted similarity provides a comprehensive measure that takes into consideration both the similarity between the vectors and the significance of the association, allowing for a more accurate assessment of the relationships between different dietary fibers or diseases within a multi‐dimensional microbial data matrix. By incorporating the weighted similarity, researchers can gain a more comprehensive understanding of the intricate connections between dietary fibers and diseases based on their microbial profiles.

(6) $${\mathrm{SIM}}_{i,j}=\frac{W_{i,j}^{S}SIM_{i,j}^{S}+W_{i,j}^{G}SIM_{i,j}^{G}+W_{i,j}^{F}SIM_{i,j}^{F}+W_{i,j}^{O}SIM_{i,j}^{O}+W_{i,j}^{C}SIM_{i,j}^{C}+W_{i,j}^{P}SIM_{i,j}^{P}}{W_{i,j}^{S}+W_{i,j}^{G}+W_{i,j}^{F}+W_{i,j}^{O}+W_{i,j}^{C}+W_{i,j}^{P}}.$$

In Equation (6), the subscript i and j represented the dietary fibers or diseases being compared, while the superscript S,G,F,O,C,P denoted the six levels in the data matrix, species, genus, family, order, class, and phylum.

To obtain the weighted *p*‐value, we utilized the standard deviation of each level as a reference to calculate a value known as the weighted deviation. We calculated the weighted deviation $\bar{\sigma }$ by taking the square root of the reciprocal of the sum of weights at the six levels. The *t*‐score was then calculated as the quotient of the weighted similarity ${\mathrm{SIM}}_{i,j}$ and the weighted deviation $\bar{\sigma }$. Lastly, we calculated the weighted *p*‐value using an unpaired two‐tailed Student's *t*‐test. To minimize false positive results, we employed the classical approach of controlling the false discovery rate (FDR) using Benjamini–Hochberg [63] method.

### Dietary fiber and disease network construction and visualization

To explore the relationships between dietary fibers, diseases, and their associated microbiota disturbances, we constructed three types of networks: dietary fiber–fiber, disease–disease, and fiber–disease. Each network was developed based on pairwise similarities calculated using the BHWA algorithm.

Dietary fiber–fiber similarity network: For the dietary fiber–fiber similarity network, where i,j∈{32fibers}, we calculated pairwise similarities between all 32 dietary fibers. At each level of analysis, we employed the adapted Equations (1)–(3) to calculate the similarity for microbiota‐based fiber i and fiber j, respectively. The weighted cosine similarity ${\mathrm{SIM}}_{i,j}\;$between fiber i and fiber j was computed using Equation (6).

Disease–disease similarity network: Similarly, for disease–disease network, where i,j∈{161diseases}, we calculated pairwise similarities between all 161 human diseases. The same set of Equations (1)–(3) was used to calculate the similarity for each disease pair. The weighted cosine similarity ${\mathrm{SIM}}_{i,j}$ between disease i and disease j was computed using Equation (6).

Fiber–disease similarity network: In the fiber–disease network, where i∈{32fibers} and j∈{123diseases}, we assessed the pairwise similarity between dietary fiber and disease. Similar to the previous networks, we applied the adapted Equations (1)–(3) to calculate the similarity for fiber i and disease j based on microbiota disturbance. The weighted cosine similarity ${\mathrm{SIM}}_{i,j}$ between fiber i and disease j was computed using Equation (6).

To ensure the credibility and clarity of the network relationship, we set the significance threshold of FDR to be less than 0.05. The networks were constructed by linking nodes (fibers or diseases) with a red line or blue line (the positive or negative similarity values obtained from the weighted analysis). These networks, visualized based on observed disturbances in the human microbiota, provide insights into the complex interrelationships among dietary fibers, diseases, and microbiota disturbances.

### Definition for *DS*

To understand the impact of diseases on microbiota across different body sites, we developed a *DS* to quantify and compare microbiota disturbances related to specific diseases. This process involved several key steps:

Body site segmentation: We categorized microbiota evidence based on the body sites where samples were collected. A total of 45 specific body sites were identified (Figure S4F). Evidence from sites with only a single disease was filtered out, leaving a data set with 39 body sites for analysis.

Multi‐level body‐site‐specific information matrix: We generated a multi‐level body‐site‐specific information matrix by hierarchically inferring microbiome information from lower to higher taxonomic levels. For each level, the relationship between human microbe m and disease i in body site was quantified using Equation (1). Taking the species level as an example:

To ensure the normalization of disturbance strength from the evidence in a specific body site, we employed the sigmoid function limiting the range of the disturbance strength $\hat{D_{m,i}^{S}}$ from −1 to 1 [30] within Equation (7). Scores close to 1 indicated an increasing trend in microbial relative abundance under disease‐related disturbance, while scores close to −1 indicated a decreasing trend.

(7) $$\hat{D_{m,i}^{S}}=\frac{2}{1+\frac{1}{e^{D_{m,i}^{S}}}}-1.$$

In Equation (7), the symbol “e” represented the mathematical constant known as the natural constant e. Here S represented the species level. The subscript i and m represented disease and microbe being compared. Thus, after one round of computation, the normalized disturbance strength at six levels could be obtained.

Disturbance score calculation: We measured the deviation between disease and health states by the Euclidean distance between two vectors, defining it as the body‐site‐specific *DS* of the disease. Then, the disturbance score ${\mathrm{DS}}_{i}$ of each disease i at a specific body site was calculated at different hierarchical levels using Equation (8).

(8) $${\mathrm{DS}}_{i}=\sqrt{\sum_{m=1}^{M^{S}}{\left(\hat{D_{m,i}^{S}}\right)}^{2}+\sum_{m=1}^{M^{G}}{\left(\hat{D_{m,i}^{G}}\right)}^{2}+\sum_{m=1}^{M^{F}}{\left(\hat{D_{m,i}^{F}}\right)}^{2}+\sum_{m=1}^{M^{O}}{\left(\hat{D_{m,i}^{O}}\right)}^{2}+\sum_{m=1}^{M^{C}}{\left(\hat{D_{m,i}^{C}}\right)}^{2}+\sum_{m=1}^{M^{P}}{\left(\hat{D_{m,i}^{P}}\right)}^{2}}.$$

where M represented the microbe count. The subscript i and m represented disease and microbe being compared. The superscript S,G,F,O,C,P denoted the six levels in the data matrix, species, genus, family, order, class, and phylum.

### Coherency determination of three‐node‐loops in network

In network analysis, three‐node loops are used to assess the coherency of relationships within a network. Three‐node loops design was based on the cycle theory in graph theory, which encompasses two fundamental forms: positive loops and negative loops (Figure S3E). In a positive cycle, the relationships between three nodes exhibit a positive directional loop (coherent). For instance, Node A is positively correlated with Node B, Node B is positively correlated with Node C, and Node C is positively correlated with Node A. In a negative cycle, the relationships between three nodes exhibit a reverse directional loop (incoherent). For example, Node A is negatively correlated with Node B, Node B is negatively correlated with Node C, and Node C is negatively correlated with Node A.

In the fiber‐fiber network and disease‐disease network, we calculated the observed ratio of the count of coherent‐loops to incoherent‐loops and conducted 10,000 random tests. In each test, we randomized the quantitative relationships between microbiota and dietary fiber or disease, and then recomputed the theoretical ratios of the count of coherent‐loops to that of incoherent‐loops. We determined the network's coherency or incoherency based on the distribution of theoretical ratio values.

### Murine model experiment design

Plantago asiatica L. seeds from Ji'an, Jiangxi (China) were used to prepare water‐soluble arabinoxylan according to the published method [64]. Female BALB/c mice (20.0 ± 2.0 g, Certificate Number SYXK (gan) 2021‐0004) were obtained from the College of Jiangxi Traditional Medicine, and the animal experiments were conducted in accordance with the Guidelines for the Care and Use of Laboratory Animals issued by the US National Institute of Health (NIH) and approved by the Animal Care Review Committee of Nanchang University, China.

In establishing murine models of inflammatory bowel disease, female BALB/c mice were subjected to acute inflammation in the colon by administering dextran sulfate sodium (DSS) in their drinking water for 11 days. Animals were quarantined for 7 days after arrival. They were kept under conditions of controlled light cycle (12‐h light and 12‐h dark) and temperature (25 ± 0.5°C). Then, they were randomized by body weight (BW) into groups 1 to 9 (Figure 5A). All groups were provided with a common basal diet according to the previous study [65]. The mice were randomized into different groups based on body weight and received either normal water or water containing DSS along with arabinoxylan administered by gavage. In establishing murine models with arabinoxylan feed, arabinoxylan was given at high or low doses. The high dose of arabinoxylan was 0.4 g/kg BW, while the low dose was 0.2 g/kg BW. Mice were given arabinoxylan at about 9:00 am every day.

We divided the nine groups of mice into three types (Figure 5A): healthy control group (labeled as C, H, and L), late‐induced group (to investigate the preventive effect of arabinoxylan; labeled as M1, HM1, and LM1), and early‐induced group (to investigate the therapeutic effect of arabinoxylan; labeled as M2, HM2, and LM2). Each type was further divided into three subgroups: basal subgroup, high‐dose arabinoxylan feed subgroup (labeled with prefix “H”), and low‐dose arabinoxylan feed subgroup (labeled with prefix “L”). Specifically, group 1 (control, C) was only given normal water for 18 days. Group 2 (high dose arabinoxylan, H) was only given arabinoxylan at a high dose for 18 days. Group 3 (low dose arabinoxylan, L) was only given arabinoxylan at a low dose for 18 days. Late‐induced group: group 4 (DSS 1, M1) was given normal water from day 1 to day 7, and then only given DSS for the remaining 11 days; group 5 (high dose arabinoxylan + DSS, HM1) was given arabinoxylan at a high dose from day 1 until the last day (day 18), and concurrently given DSS from day 8 until the last day (day 18); group 6 (low dose arabinoxylan + DSS, LM1) was given arabinoxylan at low dose from day 1 until the last day (day 18), and concurrently given DSS from day 8 until the last day (day 18). Early‐induced group: Group 7 (DSS 2, M2) was only given DSS from day 1 to day 11, and then only given normal water for the remaining 7 days; group 8 (DSS + high dose arabinoxylan, HM2) was given arabinoxylan at high dose from day 1 until the last day (day 18), and concurrently given DSS from day 1 to day 11; group 9 (DSS + low dose arabinoxylan, LM2) was given arabinoxylan at low dose from day 1 until the last day (day 18), and concurrently given DSS from day 1 to day 11.

At the end of the experiment, the mice were killed, and their organs and colon tissue samples were collected for analysis of microbiota. Different groups were categorized as either arabinoxylan, the preventative effect, or therapeutic effect groups. Furthermore, the healthy control group and the early‐induced group had 12 mice per group (6 mice in each group were killed at day 11 when DSS treatment was finished, and the rest were killed at the end of the experiment like other groups), while the late‐induced group had 6 mice per group.

### Macroscopic assessment and histopathological evaluation

As colon shortening and fecal bleeding were regarded as key indicators of colonic inflammation, we evaluated colonic damage by measuring colon length, stool consistency (0 points were for normal feces, 1.5 points for pasty feces, and 3 points for liquid feces), and fecal occult blood (0 points for negative hemoccult, 1.5 points for a positive hemoccult, and 3 points for gross bleeding).

One part of the colon was used for histology analysis. 20% of the distal third of the colon was stored in a formalin solution. After that, histological scoring for hematoxylin and eosin‐stained tissues was carried out: inflammation (0 to 3 for none to severe), extent (0 to 3 for none to transmural), and crypt damage (0 to 3 for none to entire crypt and epithelium lost). A total score (0 to 9) was obtained by summing these three scores.

### RNA isolation and qPCR assay for colon tissue

Colon tissue samples preserved in RNAlater were homogenized, and total RNA was extracted from each sample using the TRIzol method. The RNA samples were further purified to remove DNA. Complementary DNA (cDNA) for quantitative PCR (qPCR) assays was synthesized from purified RNA (1 μg) using random hexamers (50 ng) and Superscript II first‐strand cDNA synthesis kit (Invitrogen).

Quantitative PCR primers for target genes were synthesized according to the published sequences. Assays were carried out on a Stratagene MX3005 thermal cycler. cDNA was diluted 10‐fold, and 1 μL of each diluted sample was added to a 25 μL reaction solution. Cycling parameters were set as follows: 3 min at 95°C; 38 cycles of 30 s at 95°C, 60 s at annealing temperature, and 30 s at 72°C; and then extension for 2 min at 72°C. GADPH, which exhibits a stable expression level, was selected as the reference gene for qPCR. We calculated the expression level of each sample in the qPCR experimental group relative to the control group using the formula 2^−ΔΔCt^, and then calculated the average of the experimental group to obtain the expression level of the target gene.

### ELISA analysis of colon tissue

For the ELISA analysis of colon tissue, a portion of the colon tissue sample was quickly placed into a stoppered tube. Deionized water was added to the tissue at a ratio of 1:9 (tissue to water), and the mixture was gently mixed for 5 min. Following this, the samples were homogenized, and the tubes were placed in an ice‐cold water bath for 15 min. The homogenized samples were then centrifuged at 4°C at 4800 *g* for 15 min. The supernatant was carefully collected and transferred to a new stoppered tube for further analysis.

Cytokine levels, including cyclooxygenase‐2 (COX‐2), interleukin‐1 beta (IL‐1β), interleukin 10 (IL‐10), inducible nitric oxide synthase (iNOS), and interleukin 6 (IL‐6), in the colon tissue were measured using Mouse Enzyme Immunoassay kits from Wuhan Boster Technology, China. Absorbance readings were taken with a Thermo Scientific Varioskan Flash spectrophotometer (Thermo Fisher Scientific) to quantify the cytokine levels in the tissue samples.

### 16S rRNA gene sequence analysis

Total DNA was extracted from colon fecal samples (200 mg of frozen colon feces) by QIAamp DNA Stool Mini Kit. The PCR reactions were conducted in triplicate using 2.5 × Master Mix (5 Prime PCR Master Mix, Qiagen), with the forward primer CCGTCAATTCMTTTGAGTTT and the reverse primer ACTCCTACGGGAGGCAGCAG, using 50 ng of purified input DNA.

PCR conditions included an initial incubation at 95°C for 2 min, followed by 30 cycles of 20 s at 95°C, 20 s at 52°C, and 1 min at 65°C. The triplicate PCR reactions were pooled, checked by gel electrophoresis, and purified using AMPure beads (Agencourt AMPure XP, Beckman Coulter). The purified samples were then combined at equimolar concentrations and sequenced using the Miseq platform.

The 16S rRNA gene sequences were processed to filter out low‐quality sequences, denoised, and analyzed using QIIME v2.0. Additionally, the data were normalized based on the abundance of each taxonomic group in the original samples.

### Web implementation

The microbiota‐based human disease and dietary fiber databank web application (mDiFiBank, https://mdifibank.org.cn/) was developed using the R programming language and was implemented within the Shiny framework to create an interactive web interface. This platform offered users a range of features, including data access, search capabilities, visualization tools, and data download options.

To support interactive network visualization, the application utilized the visNetwork package, which was compatible with Shiny and leveraged the vis.js JavaScript library. By integrating HTML widgets, mDiFiBank provided dynamic and interactive visualizations of networks, which enhanced the user experience and facilitated the exploration of complex relationships within the microbiota‐disease and dietary fiber landscape.

## AUTHOR CONTRIBUTIONS

**Xin Zhang**: Methodology; conceptualization; writing—original draft; data curation; investigation; visualization; software; formal analysis. **Huan Liu**: Funding acquisition; writing—original draft; writing—review and editing; validation; resources. **Yu Li**: Software; methodology; funding acquisition. **Yanlong Wen**: Validation. **Tianxin Xu**: Visualization. **Chen Chen**: Visualization. **Shuxia Hao**: Visualization. **Jielun Hu**: Funding acquisition; validation; project administration; supervision; conceptualization; resources. **Shaoping Nie**: Supervision; writing—review and editing; project administration; funding acquisition; conceptualization; resources. **Fei Gao**: Supervision; funding acquisition; writing—review and editing; conceptualization. **Gengjie Jia**: Writing—review and editing; supervision; project administration; funding acquisition; conceptualization; methodology.

## CONFLICT OF INTEREST STATEMENT

The authors declare no conflicts of interest.

## ETHICS STATEMENT

All animal experiments were performed under the Guidelines for Care and Use of Laboratory Animals of the National Institutes of Health and were approved (No. NCULAE‐20221030003) by the Experimental Animal Care and Use Committee of Nanchang University.

## Supporting information


**Figure S1.** Workflow of Bio‐taxonomic Hierarchy Weighted Aggregation (BHWA) algorithm.
**Figure S2.** Flowchart for collecting the microbiota‐related evidence.
**Figure S3.** Features of dietary fiber‐microbe evidence.
**Figure S4.** Features of disease‐microbe evidence.
**Figure S5.** Phylogenetic tree view of disease similarity clusters.
**Figure S6.** Analysis of human disease similarity based on the human microbiota disturbance data (upper) and etiology information data (lower).
**Figure S7.** Correlation between microbiota‐based and etiology‐based disease similarity.
**Figure S8.** Disturbance score and features of microbiota disturbance under different diseases.
**Figure S9.** Features of the dietary fiber‐disease similarity distribution.
**Figure S10.** Comparison of microbe compositions and cytokine levels on murine models of inflammatory bowel disease.

**Table S1.** List of disease disturbance score (*DS*) at different body sites.
**Table S2.** Statistical analysis of disease disturbance scores (*DS*) across 29 body sites.
**Table S3.** The similarity of microbial disturbance among asthma and T2D in different hierarchical classifications.
**Table S4.** The similarity of microbial disturbance at six levels among Crohn's disease, ulcerative colitis and growth hormone deficiency.
**Table S5.** Abbreviations and full names of various dietary fibers.
**Table S6.** Disease names from the literature evidence dataset mapped to their corresponding etiological grouping categories.

## Data Availability

The data that support the findings of this study are available from the corresponding author upon reasonable request. We provided a Code Ocean capsule including executable programming scripts, input and output data at https://doi.org/10.24433/CO.1754986.v1. Code Ocean is a cloud‐based platform designed to enable reproducible research and collaboration. It allows users to create, share, and run computational environments, providing a containerized space where code can be executed alongside its dependencies, data, and results. Certainly, we have also released the code and data on GitHub at https://github.com/zhangxiner2/Cumulative-Profiling-of-Microbiota-Disturbance. Supplementary materials (figures, tables, graphical abstract, slides, videos, Chinese translated version, and update materials) may be found in the online DOI or iMeta Science http://www.imeta.science/.
