# Supplementary material for: Linking dietary fiber to human malady through cumulative profiling of microbiota disturbance
Source: Imeta. 2025 Feb 19;4(1):e70004. doi: 10.1002/imt2.70004 (PMC11865338; doi:10.1002/imt2.70004)
Supplement: Supplementary file 1 — Figure S1. Workflow of Bio‐taxonomic Hierarchy Weighted Aggregation (BHWA) algorithm. Figure S2. Flowchart for collecting the microbiota‐related evidence. Figure S3. Features of dietary fiber‐microbe evidence. Figure S4. Features of disease‐microbe evidence. Figure S5. Phylogenetic tree view of disease similarity clusters. Figure S6. Analysis of human disease similarity based on the human microbiota disturbance data (upper) and etiology information data (lower). Figure S7. Correlation between microbiota‐based and etiology‐based disease similarity. Figure S8. Disturbance score and features of microbiota disturbance under different diseases. Figure S9. Features of the dietary fiber‐disease similarity distribution. Figure S10. Comparison of microbe compositions and cytokine levels on murine models of inflammatory bowel disease. [file IMT2-4-e70004-s001.docx]

Supporting information to

**Linking dietary fiber to human malady through cumulative profiling of microbiota disturbance**

**Running title:** Cumulative profiling: linking fiber, microbiota to human malady

Xin Zhang^1#^, Huan Liu^2#^, Yu Li^3^, Yanlong Wen^2^, Tianxin Xu^1^, Chen Chen^1^, Shuxia Hao^1^, Jielun Hu^2^*, Shaoping Nie^2^*, Fei Gao^1,4^*, Gengjie Jia^1^*

^1^Genome Analysis Laboratory of the Ministry of Agriculture and Rural Affairs, Agricultural Genomics Institute at Shenzhen, Chinese Academy of Agricultural Sciences, Shenzhen 518120, China

^2^State Key Laboratory of Food Science and Resources, China-Canada Joint Lab of Food Science and Technology (Nanchang), Key Laboratory of Bioactive Polysaccharides of Jiangxi Province, Nanchang University, Nanchang 330047, China

^3^Department of Computer Science and Engineering, The Chinese University of Hong Kong, Hong Kong 999077, China

^4^Comparative Pediatrics and Nutrition, Department of Veterinary and Animal Sciences, Faculty of Health and Medical Sciences, University of Copenhagen, Copenhagen DK-2100, Denmark

^#^ These authors contributed equally: Xin Zhang, Huan Liu

^*^Corresponding authors: [hujielun@ncu.edu.cn](mailto:hujielun@ncu.edu.cn) (Jielun Hu); [spnie@ncu.edu.cn](mailto:spnie@ncu.edu.cn) (Shaoping Nie); [flys828@gmail.com](mailto:flys828@gmail.com) (Fei Gao); [jiagengjie@caas.cn](mailto:jiagengjie@caas.cn) (Gengjie Jia)


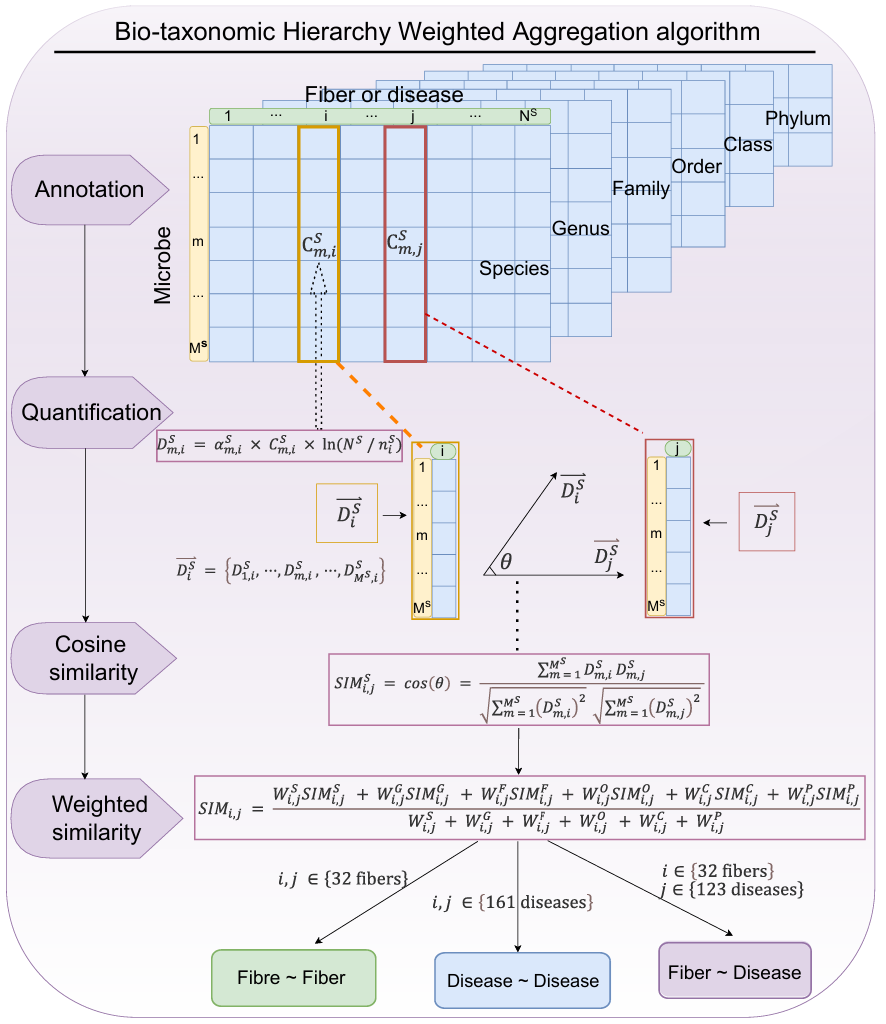


**Figure S1 Workflow of Bio-taxonomic Hierarchy Weighted Aggregation (BHWA) algorithm.** “Annotation”: Generating a multi-level microbe evidence matrix by hierarchically inferring microbe information from lower to higher taxonomic levels that spanned species, genus, family, order, class, and phylum. “Quantification”: Calculation of the relationship between microbiota and dietary fibres (or diseases) at each taxonomic level using the evidence matrix. “Cosine similarity calculation”: Measurement of pairwise cosine similarities for dietary fibres (or diseases) at each taxonomic level, with significance-based weight. “Weighted similarity determination”: Calculation of weighted similarity by integrating cosine similarities and weights across the six taxonomic levels.


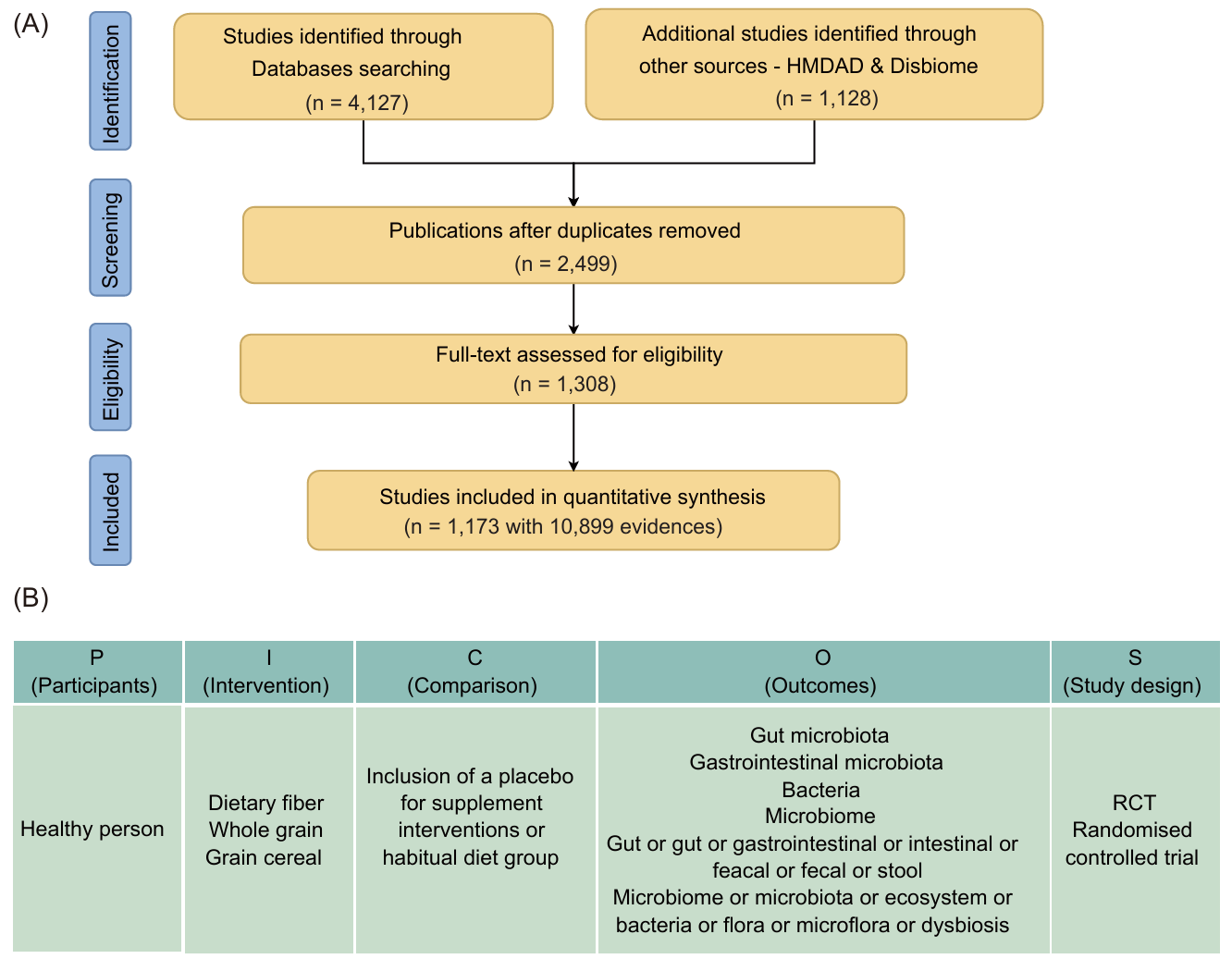


**Figure S2 Flowchart for collecting the microbiota-related evidence.** (A) The literature screening process for the related microbiota studies. The evidence of dietary-fiber-microbe database was collected from PubMed, MEDLINE, Web of Science, and Embase via a series of screening criteria and de-duplication treatments, while the evidence of disease-microbe database was collected from PubMed, Human Microbe-Disease Association Database (HMDAD), and Disbiome. This process consisted of a series of screening criteria and de-duplication treatments to ensure the quality and reliability of the data. Eligible studies included randomized controlled trials (RCTs), cluster RCTs, or quasi-RCTs, with the primary outcome being changes in microbial composition at the end of the intervention period. (B) Key words used for literature searches related to dietary fiber and microbe. The search was based on the PICOS principle, and these keywords were used for initial screening of relevant literature types in major databases: PubMed, MEDLINE, Web of Science, and Embase.


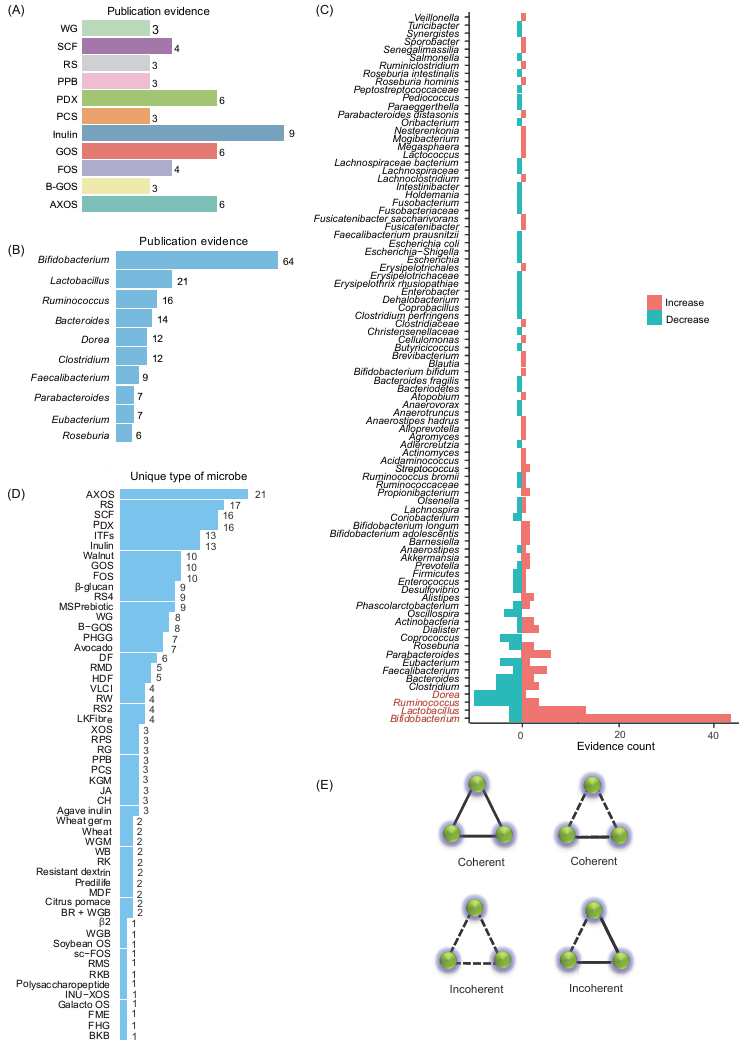


**Figure S3 Features of dietary fiber-microbe evidence.** (A) Evidence counts for top 11 dietary fibers in the dietary-fiber-microbe database. The Y-axis indicated the types of dietary fibers, and the height of each bar reflected the number of publications providing evidence about these top 11 fibers. (B) Evidence counts for top 10 microbes in the dietary-fiber-microbe database. The Y-axis represented different types of disturbed microbes, with the height of each bar indicating the number of publications providing evidence about these top 10 microbes. (C) Evidence counts supporting directional changes of microbial abundance in response to dietary fiber. The height of the bars represented the number of publications supporting either an increase or a decrease in microbial abundance following fiber intervention compared to a normal diet, with the Y-axis indicating the associated microbe with the intervention of dietary fiber. (D) Frequency distribution of corresponding microbes associated with all dietary fibers. The X-axis referred to unique count of disturbed microbes via dietary fibers intervention, while the Y-axis represented different types of dietary fiber in dietary-fiber-microbe database. Abbreviations for dietary fibers were used, with full names provided in Table S5. (E) Types of three-node-loops in network. Solid lines denoted positive similarity relationships between nodes, while dashed lines indicated negative similarity relationships. The coherent loops, characterized by an even number of negative links, were contrasted with incoherent loops, which have an odd number of negative links.


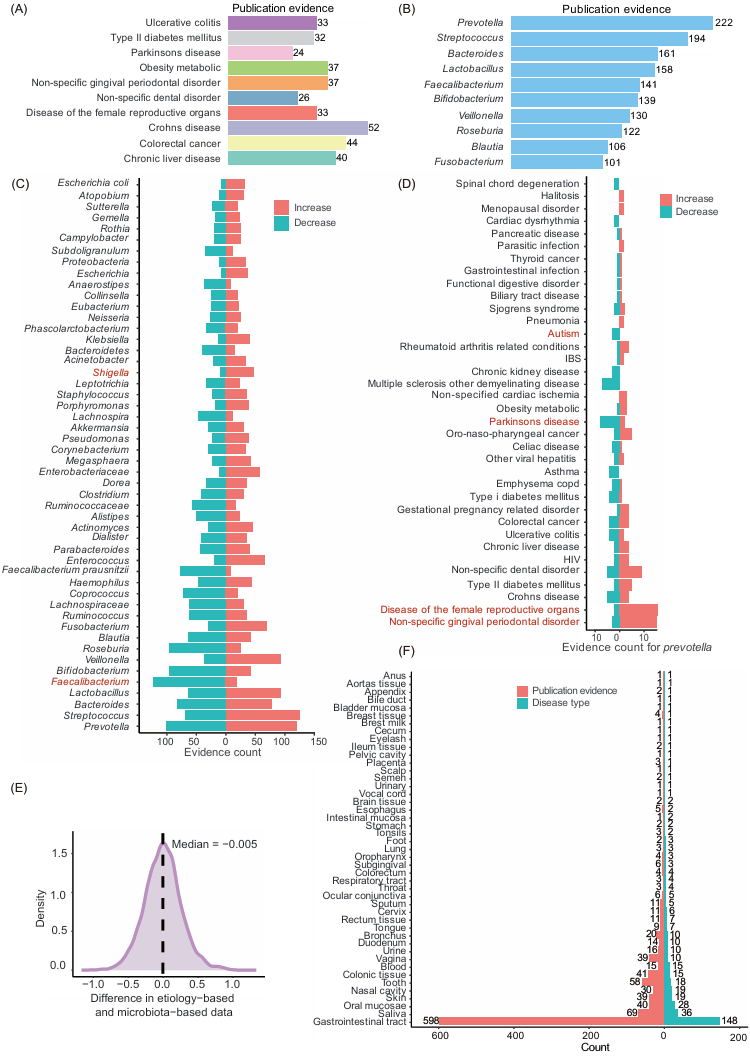


**Figure S4 Features of disease-microbe evidence.** (A) Evidence counts for top 10 diseases in the disease-microbe database. The Y-axis represented different human diseases, with the height of each bar indicating the number of publications that provided evidence. (B) Evidence counts for top 10 microbes in the disease-microbe database. The Y-axis listed various disturbance microbes, and the height of each bar showed the number of publications providing evidence about these top 10 microbes. (C) Evidence counts supporting directional changes of microbial abundance in response to disease. Bar height indicated the number of publications supporting an increase or a decrease of microbial abundance in patients compared to healthy people. The Y-axis represented the top 50 microbe associations with diseases in the human disease-microbe database. (D) Evidence counts supporting directional changes of *Prevotella* response to diseases. The Y-axis listed diseases associated with *Prevotella*, while the bar heights indicated the number of publications reporting increases or decreases in *Prevotella* relative abundance in patients compared to healthy people. (E) Distribution of differences between etiology-based and microbiota-based similarities. The X-axis represented the difference (delta) between etiology-based similarities and microbiota-based similarities, while the Y-axis showed the density of these differences. The median value reflected the central tendency of these deltas. (F) Overview of data distribution across 45 specific body sites. The X-axis represented the evidence count, either in terms of the number of publications or the number of disease types, while the Y-axis listed the 45 body sites. The red bars denoted the number of publications that focused on studying these body sites, while the blue bars indicated the number of diseases associated with each site.


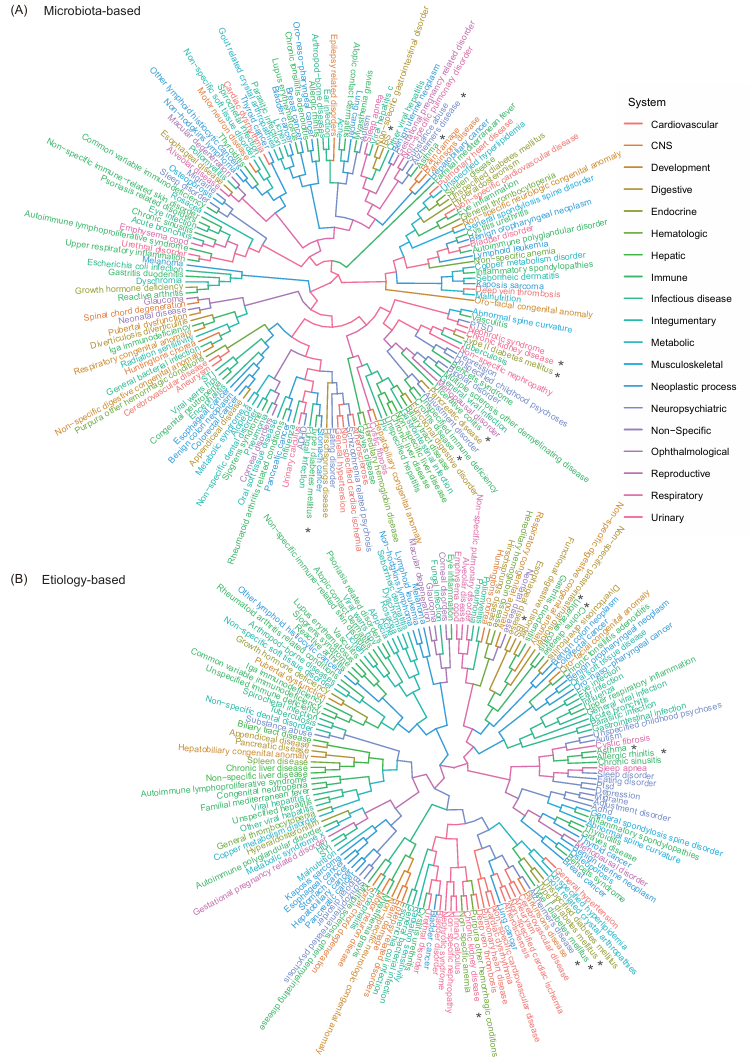


**Figure S5 Phylogenetic tree view of disease similarity clusters.** (A) Phylogenetic tree of disease clusters based on microbiota disturbance data. This tree displayed the clustering of diseases according to their microbiota disturbance profiles. The branches and diseases were color-coded by general disease categories, as indicated in the legend. CNS, central nervous system. (B) Phylogenetic tree of disease clusters based on etiology information data. This tree showed disease clustering based on etiological data. Like (A), branches and diseases were color-coded by general disease categories as per the legend. Diseases of interest discussed in the text were also marked with stars.


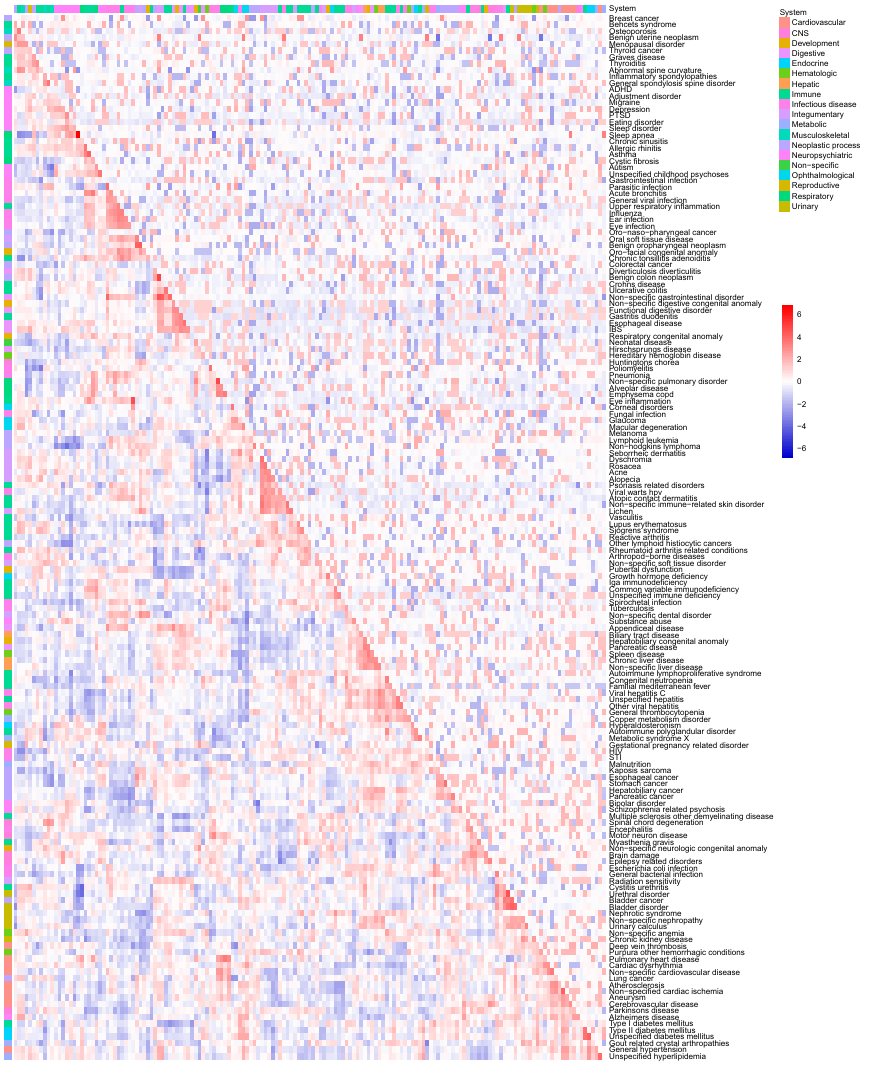


**Figure S6 Analysis of human disease similarity based on the human microbiota disturbance data (upper) and etiology information data (lower).** Cells were filled in color gradients from red to blue, representing positive to negative associations between corresponding microbiota-based (lower) and etiology-based (upper) human diseases. The X-axis and Y-axis were colored by different disease systems, as indicated in the legend. Each row and column corresponded to a specific disease, with diseases sorted in the same order and symmetrically distributed along the diagonal. Only the disease names in the rows were displayed, while the column disease names were hidden. CNS, central nervous system.


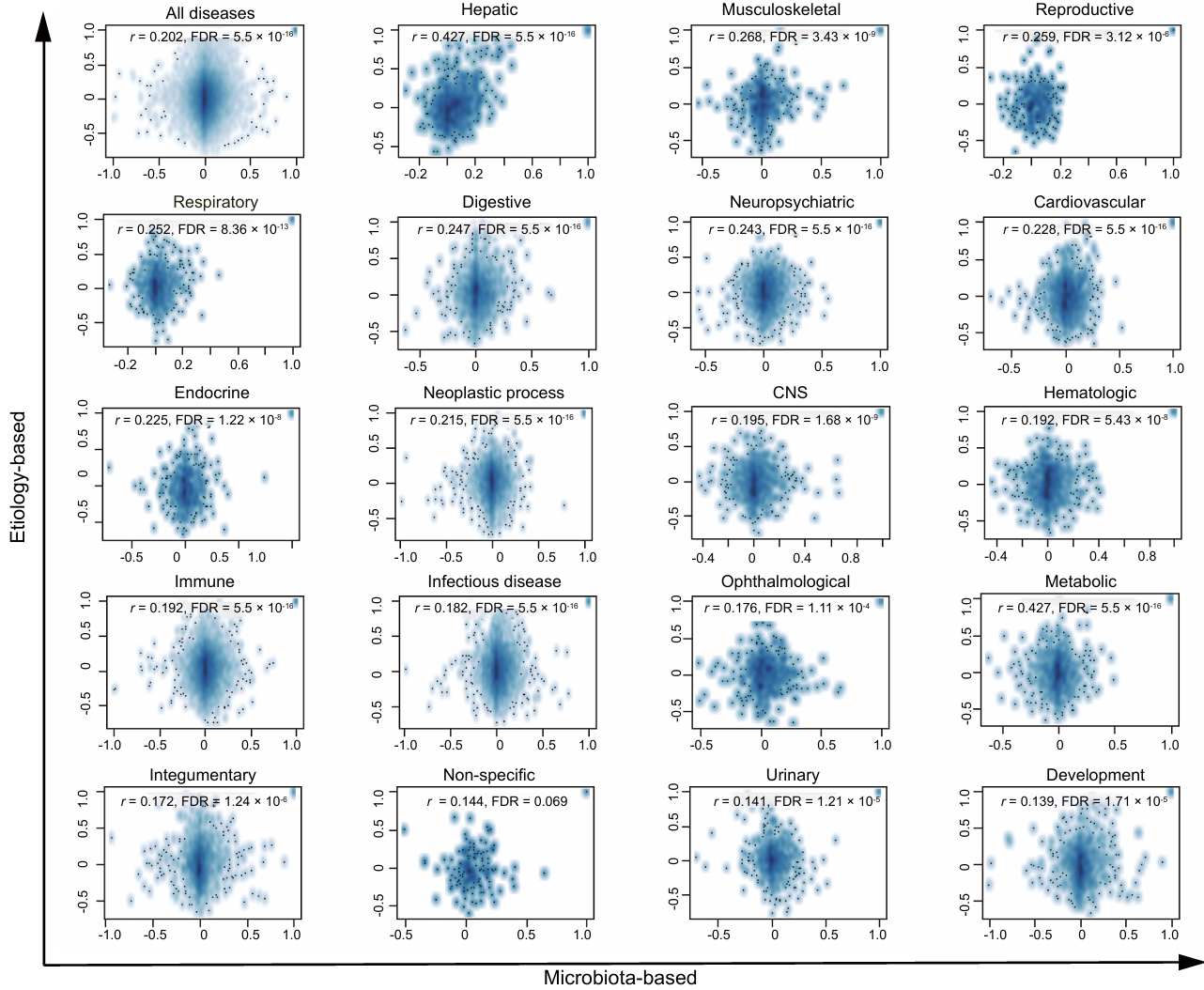


**Figure S7 Correlation between microbiota-based and etiology-based disease similarity**. The X-axis represented the pairwise disease similarity based on microbiota disturbance, while the Y-axis showed the similarity based on etiology information for all 161 disease groups. The first analysis aimed to illustrate the general correlation between microbiota-based and etiology-based disease similarities. The residual section delved into the correlation within 19 different disease systems, using both microbiota disturbance data and etiology information. It highlighted how disease similarity correlates within specific systems rather than across all diseases. The symbol “*r*” represented the Pearson’s correlation coefficient, and the Benjamini-Hochberg method was employed to control the false discovery rate (FDR).


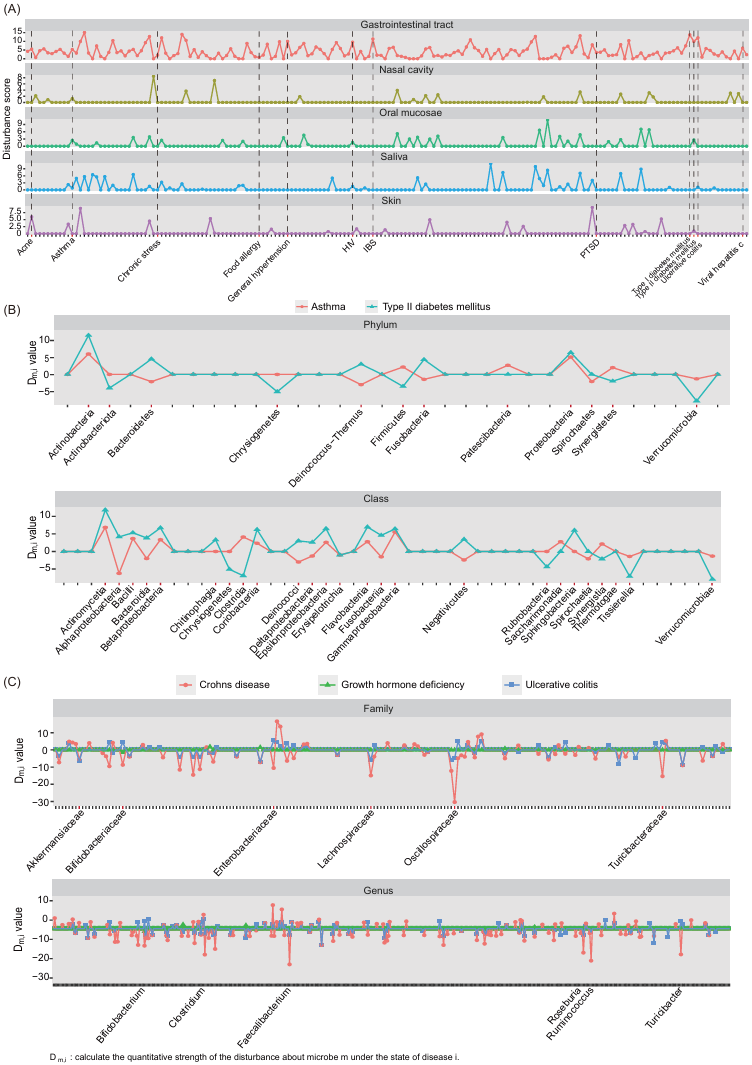


**Figure S8 Disturbance score and features of microbiota disturbance under different diseases.** (A) Body-site-specific diseases disturbance score (*DS*) in top five ranked sample sources. The X-axis represented several common disease types, and the Y-axis displayed the disease disturbance score computed based on microbiota changes at specific body sites. Higher scores indicated a greater impact of the disease on the microbiota. (B) Directional changes of microbiota disturbance at the phylum and class levels in individuals with asthma and type 2 diabetes mellitus. The X-axis showed microbiota with noted disturbance changes, while the Y-axis labeled as $D_{m,i}$ represented the quantitative strength of microbiota disturbance for each microbe under the disease state. (C) Directional changes of microbiota disturbance at the family and genus levels in individuals with Crohn’s disease, ulcerative colitis, and growth hormone deficiency. The X-axis displayed the six most disturbed microbes at these taxonomic levels, and the Y-axis$D_{m,i}$ showed the strength of disturbance for each microbe under the disease state.


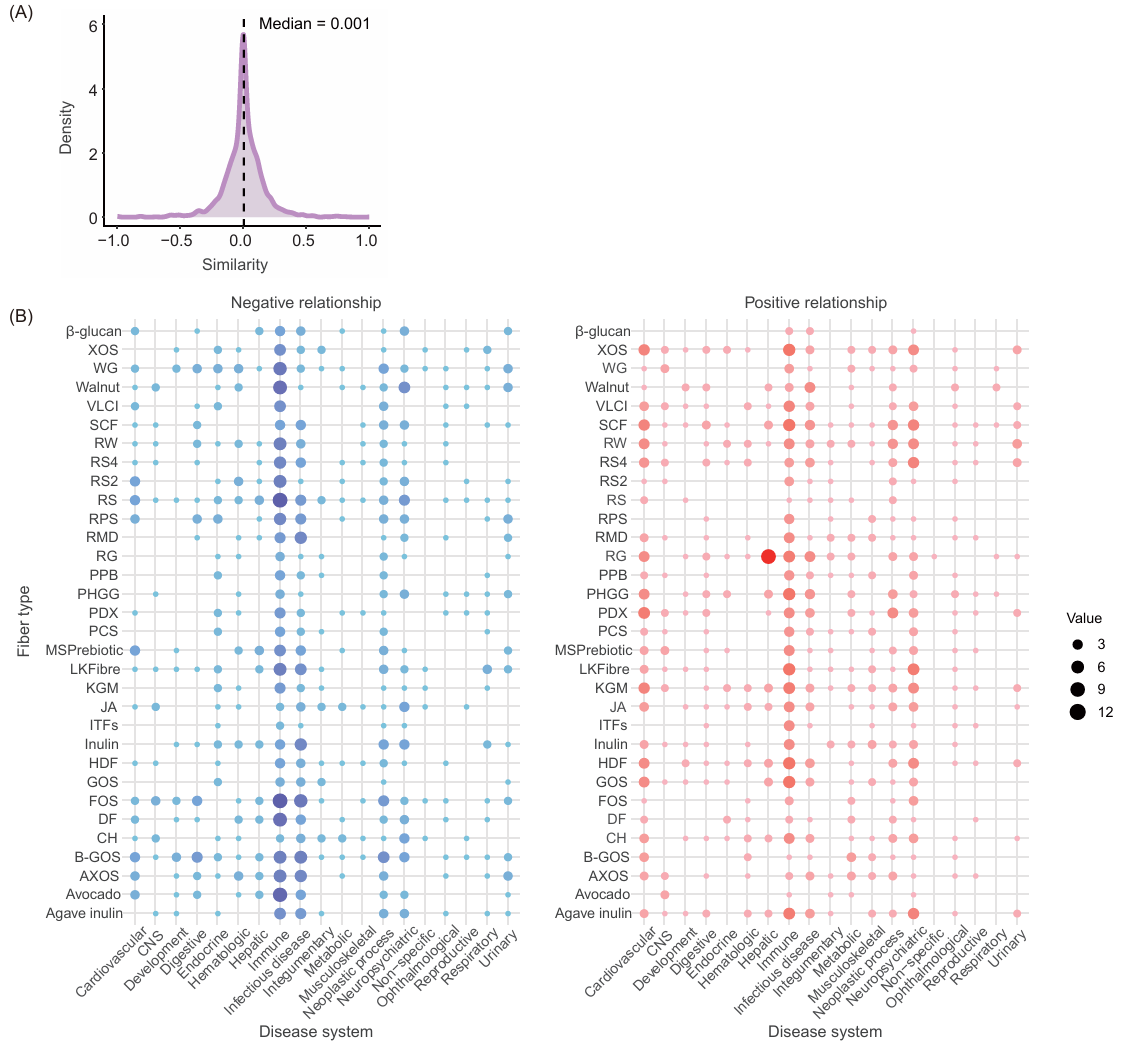


**Figure S9 Features of the dietary fiber-disease similarity distribution.** (A) The overall distribution of fiber-disease similarities. The X-axis represented the degree of similarity between each dietary fiber and disease, while the Y-axis showed the density of these similarities. The median value was calculated from the distribution. (B) The pattern between dietary fiber and disease system. The X-axis represented the classification of diseases, while the Y-axis represented the types of fiber. The value represents the number of disease systems that have negative (blue dots) or positive (red dots) microbial disturbance patterns with dietary fiber. The larger the value, the larger the points will be, as shown in the legend. Abbreviations for dietary fibers were used, with full names provided in Table S5.


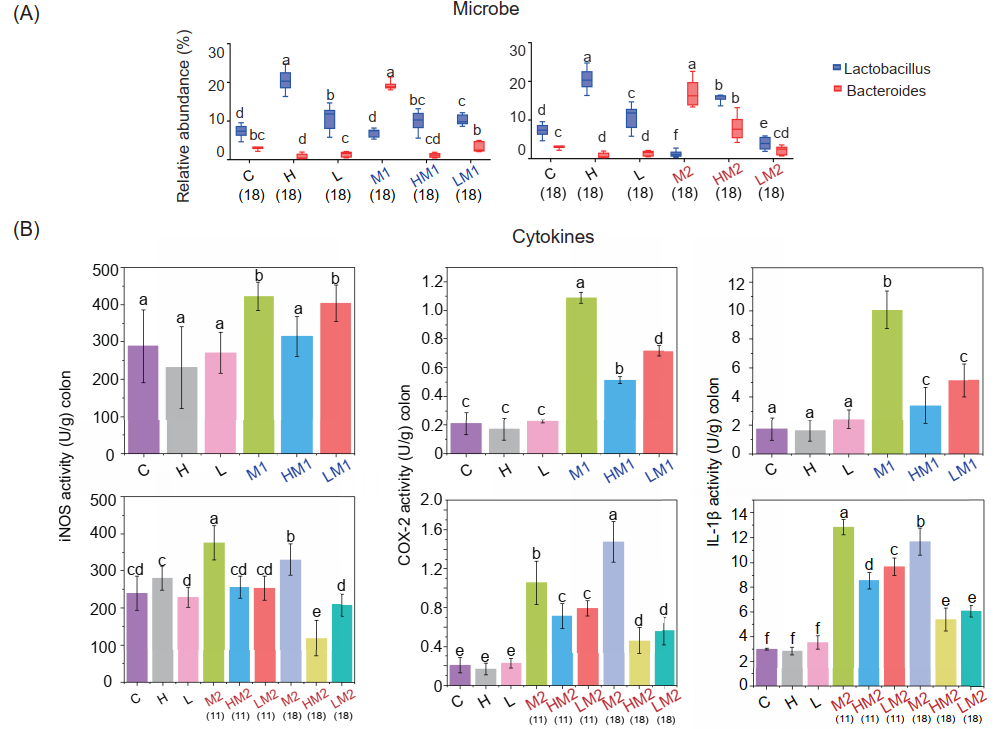


**Figure S10 Comparison of microbe compositions and cytokine levels on murine models of inflammatory bowel disease.** (A) The relative abundance of microbes at the genus level between control and late-induced (upper) and early-induced groups (lower) on day 18. The X-axis represented different mouse groups, while the Y-axis showed the relative abundance of disturbed microbes due to arabinoxylan intervention. Different microbial abundances were color-coded with error bars (as shown in legend). (B) Cytokines secretion comparison between control and late-induced (upper) and early-induced groups(lower). The X-axis represented different mouse groups, while the Y-axis showed inducible nitric oxide synthase (iNOS), inducible cyclooxygenase-2 (COX-2), and interleukin-1 beta (IL-1β) levels. Bar heights and error bars indicated the secretion amount of these cytokines in various groups. The X-axis indicated three different mice groups: Healthy control group marked with black font (C, control group; H, high dose of arabinoxylan intervention group; L, low dose of arabinoxylan intervention group), late-induced group marked with blue font (for investigating the preventative effect of arabinoxylan; labelled as M1, HM1, and LM1), and early-induced group marked with red font (for examining the therapeutic effect of arabinoxylan; labelled as M2, HM2, and LM2). Please refer to Figure 5A for the detailed information. Letter “a”, “b”, “c”, and others above the bars denoted statistical significance between groups, with identical letters indicating no significant difference and different letters indicating significant differences. The numbers in parentheses denoted the day of measurement.
